# Genome-wide association mapping of partial resistance to *Aphanomyces euteiches* in pea

**DOI:** 10.1186/s12864-016-2429-4

**Published:** 2016-02-20

**Authors:** Aurore Desgroux, Virginie L’Anthoëne, Martine Roux-Duparque, Jean-Philippe Rivière, Grégoire Aubert, Nadim Tayeh, Anne Moussart, Pierre Mangin, Pierrick Vetel, Christophe Piriou, Rebecca J. McGee, Clarice J. Coyne, Judith Burstin, Alain Baranger, Maria Manzanares-Dauleux, Virginie Bourion, Marie-Laure Pilet-Nayel

**Affiliations:** INRA, UMR IGEPP 1349, Institut de Génétique et Protection des Plantes, Domaine de la Motte au Vicomte, BP 35327, 35653 Le Rheu Cedex, France; INRA, UMR 1347 Agroécologie, 17 rue de Sully, 21065 Dijon Cedex, France; GSP, Domaine Brunehaut, 80200 Estrées-Mons Cedex, France; PISOM, UMT INRA/Terres Inovia, UMR IGEPP 1349, Domaine de la Motte au Vicomte, BP 35327, 35653 Le Rheu Cedex, France; Terres Inovia, 11 rue de Monceau, CS 60003, 75378 Paris Cedex, France; INRA, Domaine Expérimental d’Epoisses, UE0115, 21110 Bretenières Cedex, France; USDA, ARS, Grain Legume Genetics and Physiology Research Unit, Pullman, WA 99164-6434 USA; USDA, ARS, Western Regional Plant Introduction Station, Washington State University, Pullman, WA 99164-6402 USA; AgroCampus Ouest, UMR IGEPP 1349 IGEPP, 65 rue de Saint Brieuc, 35042 Rennes Cedex, France; Present Address: Nestlé R&D Center Tours, 101 Avenue Gustave Eiffel, 37097 Tours Cedex 2, France; Present Address: Chambre d’Agriculture de l’Aisne, 1 rue René Blondelle, 02007 Laon Cedex, France

**Keywords:** Root rot, Plant disease resistance, GWAS, Pea (*Pisum sativum*), Quantitative trait loci, Marker haplotype, Candidate genes

## Abstract

**Background:**

Genome-wide association (GWA) mapping has recently emerged as a valuable approach for refining the genetic basis of polygenic resistance to plant diseases, which are increasingly used in integrated strategies for durable crop protection. *Aphanomyces euteiches* is a soil-borne pathogen of pea and other legumes worldwide, which causes yield-damaging root rot. Linkage mapping studies reported quantitative trait loci (QTL) controlling resistance to *A. euteiches* in pea. However the confidence intervals (CIs) of these QTL remained large and were often linked to undesirable alleles, which limited their application in breeding. The aim of this study was to use a GWA approach to validate and refine CIs of the previously reported Aphanomyces resistance QTL, as well as identify new resistance loci.

**Methods:**

A pea-Aphanomyces collection of 175 pea lines, enriched in germplasm derived from previously studied resistant sources, was evaluated for resistance to *A. euteiches* in field infested nurseries in nine environments and with two strains in climatic chambers. The collection was genotyped using 13,204 SNPs from the recently developed GenoPea Infinium® BeadChip.

**Results:**

GWA analysis detected a total of 52 QTL of small size-intervals associated with resistance to *A. euteiches,* using the recently developed Multi-Locus Mixed Model. The analysis validated six of the seven previously reported main Aphanomyces resistance QTL and detected novel resistance loci. It also provided marker haplotypes at 14 consistent QTL regions associated with increased resistance and highlighted accumulation of favourable haplotypes in the most resistant lines. Previous linkages between resistance alleles and undesired late-flowering alleles for dry pea breeding were mostly confirmed, but the linkage between loci controlling resistance and coloured flowers was broken due to the high resolution of the analysis. A high proportion of the putative candidate genes underlying resistance loci encoded stress-related proteins and others suggested that the QTL are involved in diverse functions.

**Conclusion:**

This study provides valuable markers, marker haplotypes and germplasm lines to increase levels of partial resistance to *A. euteiches* in pea breeding.

**Electronic supplementary material:**

The online version of this article (doi:10.1186/s12864-016-2429-4) contains supplementary material, which is available to authorized users.

## Background

Research interest in polygenic resistance to plant diseases has increased worldwide in the past ten years to meet the challenge of sustainable agriculture. Recommendations to reduce chemical inputs and the frequent breakdown of major resistance genes in plants have encouraged the integration of polygenic resistance into cultivars of many crops. However, breeding strategies for polygenic resistance, which is controlled by many genes, have not been as widely developed as for monogenic or oligogenic resistance (controlled by one or few genes, respectively) [1]. Breeding schemes for polygenic traits are costly and time-consuming. The effects of Quantitative Trait Loci (QTL) controlling resistance are not always conserved in different genetic backgrounds and environments and markers tightly linked to resistance loci have also often been lacking. Further research is needed to validate QTL effects, reduce their confidence intervals (CIs) and identify their underlying causal genes [2, 3], to encourage and optimize QTL use in breeding [4].

The identification of plant resistance QTL has broadly been explored using linkage mapping populations derived from crosses between two parental genotypes [1]. With the decrease in genotyping costs and the massive development of markers in the recent past years, genome-wide association (GWA) studies, are becoming common approaches to detect natural variation underlying complex traits, especially polygenic resistance to major diseases, in a large range of crop species [5–7], including legumes [8, 9]. The advantages of GWA studies compared to bi-parental linkage mapping include access to wider genetic diversity, higher recombination rates due to the evolutionary history of the species, and thus substantially refined genomic regions associated with trait variations [5, 10]. Accurate marker density for GWA studies depends on the rate of linkage disequilibrium (LD) decay and should be higher in species with a rapid LD decay (a few kb, such as in maize) than in those with a slow LD decay (~100 kb, such as in rice) [5]. Segura et al. [11] recently proposed a Multi-Locus Mixed Model (MLMM) approach, in order to improve GWA studies precision and power of detection, and it was successfully applied [12]. The reduction of CIs of genomic regions associated with traits of interest, opened the possibility of identifying haplotypes for marker assisted selection (MAS) [8, 13] and pinpointing interesting candidate genes underlying QTL [14, 15]. However, the GWA approach has also been reported to have poor power to detect rare alleles associated with the trait of interest, leading to missing heritability [5, 16], and complementarity between linkage and GWA approaches has been underlined [7, 17]. Multi-parental designs, including Nested Association Mapping (NAM) [18, 19], Multi-parent Advanced Generation Inter-Cross (MAGIC) [20, 21] and breeding line populations [22, 23] were proven to efficiently increase power of GWA studies to detect rare variants, for which rates are increased by selection of rare-allele-carrier parental lines [7, 16].

Dry pea (*Pisum sativum*) is the third most important pulse crop worldwide [24], for which yield has been unstable for the past decades, mainly due to biotic and abiotic stresses. One of the most damaging biotic stresses of peas is Aphanomyces root rot due to *Aphanomyces euteiches* [25]. The soil-borne root pathogen, first described in 1925 [26], has been mainly reported as a yield limiting factor in the United States of America (USA) and Europe for more than twenty years [27–29], and more recently in Canada [30]. Two main pathotypes of *A. euteiches* were described by Wicker and Rouxel [28], including pathotype I predominant in France and pathotype III detected in some regions of the USA (Onfroy et al., personal communication). Both pathotypes cause honey brown necrosis symptoms on pea roots and epicotyls, resulting in dwarfism, foliage yellowing and then death of plants in highly infested fields. Increasing yield loss due to *A. euteiches* in dry and green pea production has been noted in Western Europe due to short crop rotations of susceptible pea varieties and the long lifespan of oospores [27]. The development of resistant cultivars has been considered as a major objective for the past two decades in France, as only prophylactic and cropping methods are available to manage the disease. Pea lines partially resistant to *A. euteiches* were identified from germplasm screening and breeding programs conducted in the USA [31–35], and more recently, from a French germplasm screening program of approximately 1900 *Pisum* lines [36]. The most resistant lines were integrated into crossing programs to develop breeding lines [37, 38], recombinant inbred lines (RILs) [39–43] and near-isogenic lines (NILs) [44]. Breeding lines with increased levels of resistance to *A. euteiches* were selected in a phenotypic recurrent selection-based breeding program developed by GSP (Groupement des Sélectionneurs de Pois Protéagineux, France) [37, 38]. RILs have also been used for discovery of Aphanomyces resistance QTL [39–41, 43]. A total of 27 meta-QTL were identified on a consensus genetic map from four RIL populations [43]. Eleven of them, corresponding to seven genomic regions, were detected on six of the seven pea linkage groups (LGs), with high consistency over locations, years, isolates and populations [43]. Marker assisted back-crossing was used to introgress each of the seven consistent genomic regions into one of the susceptible RIL parents and two main spring and winter pea varieties. The resulting NILs were used to validate individual or combined major resistance QTL effects [44]. Lavaud et al. [44] considered large QTL intervals for NIL creation, which brought undesirable morphological (coloured flowers, normal leaves) or developmental (late flowering) alleles linked to resistance alleles at several QTL.

Massive numbers of Single-Nucleotide Polymorphism (SNP) markers were recently developed from whole genome cDNA (coding deoxyribonucleic acid) [45–47] or genomic sequencing of pea lines [48, 49]. A GenoPea Infinium® BeadChip was developed by Tayeh et al. [49], containing 13,204 SNPs, all located in gene-context sequences. Pea diversity panels, especially the USDA (United States Department of Agriculture) core collection and the INRA (Institut National de la Recherche Agronomique) reference collection, were used to determine associations between low to medium density genetic markers (137–1233) and traits of interest [9, 50–52]. However, only a few sources of resistance to *A. euteiches* were identified in these collections (Pilet-Nayel et al., unpublished data), as was also found in larger *Pisum* screening programs for Aphanomyces resistance [34, 36].

The aim of this study was to validate and refine the CIs of previously reported Aphanomyces resistance QTL, as well as identifying new resistance loci, using a GWA approach. A novel panel, enriched in pea lines partially resistant to *A. euteiches* from gene pools previously studied, was designed including breeding and germplasm lines [37, 38, 43]. The recent GenoPea Infinium® BeadChip was used for high-density SNP genotyping of the collection [49]. The GWA study detected SNPs and LD blocks associated with Aphanomyces resistance from data collected in nine field environments and two strains under controlled conditions. The genomic positions and CIs of resistance loci detected by GWA study were compared to those previously identified by linkage analysis [41, 43]. The GWA study also identified loci associated with morphological and developmental traits, in order to analyse their linkages with Aphanomyces resistance loci. The GWA study allowed marker haplotypes and putative candidate genes to be identified, for future pyramiding of resistance alleles in breeding and to investigate the molecular basis of polygenic resistance.

## Methods

### Plant material

A collection of 175 *Pisum sativum* lines, referred to as the “pea-Aphanomyces collection", was used for association mapping. It includes cultivars, breeding lines and germplasm lines, supplied by public and private programs in legume genetics research and breeding (Additional file 1):

(i) One hundred and one breeding lines, partially resistant or susceptible to *A. euteiches*, originating from the Aphanomyces recurrent selection-based breeding program conducted by GSP [37, 38]. The breeding scheme included four crossing programs (namely AeA95, AeB97, AeC98 and AeD99), established from 1995 to 1999. Parental lines of the crosses included partially resistant germplasm lines confirmed in French field conditions (*PI180693*, *90*–*2131* and *552*) [31–34] and dry spring or winter pea cultivars with good agronomic values but susceptible to the pathogen [37, 38]. Simple or double crosses between multiple parents were designed in the AeA95, AeB97 and AeC98 programs.. The best F_1_ hybrids from the three programs were selected based on resistance levels of their inbred progenies (F_5_-F_6_). The selected F_1_ hybrids were then inter-crossed in the AeD99 program which was divided into two parts. The first part (AeD99QU) was generated from two crosses and aimed at developing pea lines with improved resistance levels for breeding purposes. The second part (AeD99OSW) was generated from three other crosses and was conducted as a divergent selection program of resistant and susceptible lines for QTL validation studies. In each of the four crossing programs, selection for resistance was conducted from F_3_ or F_4_ generations, alternatively in growth chamber and in French field nurseries. Resistance levels and agronomic values of the best F_7_ and F_8_ lines were then assessed in infested field trials and in healthy field conditions.
(ii) Twenty RILs from INRA and USDA research programs selected for Aphanomyces resistance and for morphological and developmental traits. These RILs included: (ii_a_) four lines from the *Baccara* x *PI180693* RIL population [41], (ii_b_) eight lines from the *Baccara* x *552* RIL population [41], and (ii_c_) eight lines from the *Dark Skin Perfection* (*DSP*) x *90-2131* RIL population [42, 43].
(iii) Eighteen parental lines of RIL populations used for QTL analysis of Aphanomyces resistance [39–41, 43], and/or of the Aphanomyces recurrent selection-based breeding program conducted by the GSP. These lines included the six pea differential genotypes defined by Wicker et al. [29] to verify disease severity and strain pathotypes in pathogenicity tests.
(iv) Twenty lines representing new sources of resistance, selected from the INRA screening program of Aphanomyces resistance sources previously conducted [36]. The new sources of resistance selected included pea breeding and germplasm lines from Aphanomyces resistance screening programs in the USA and from collections of genetic resource centers in France, the Netherlands, Russia and the USA (Pilet-Nayel et al., unpublished).
(v) Sixteen spring or winter pea varieties currently or formerly grown in Europe.

The pea-Aphanomyces collection thus combined different genetic sources of resistance, especially originating from three major resistant pea lines effective in France (*90*–*2131*, *552* and *PI180693*) and analysed in previous QTL studies [43]. The collection included plant material described both for different end-uses (food, feed or fodder peas) and for different sowing times (spring and winter peas). Morphological descriptions (seed type, foliage type and flower colour) and available pedigree information of the lines are presented in Additional file 1.

### Phenotyping

The collection was evaluated for Aphanomyces resistance in inoculated controlled conditions (CC) and infested fields. In CC, resistance tests were performed in 2009 on all the lines but the 20 RILs with two pure-culture reference strains of *A. euteiches* described in [41], *i.e.,* the French RB84 strain from pathotype I and the American Ae109 strain from pathotype III. CC resistance tests were also conducted in 2013 on all the lines with the RB84 strain only. The experiments used a modified version of the standardized test developed by Moussart et al. [54], as described in [41]. The pea lines were evaluated for resistance to each strain, using a randomised complete block design with four and three blocks in 2009 and 2013 tests, respectively, and five seeds per block sown in a pot. Resistance tests were conducted in a growth chamber (25 °C for 16h-day, 23 °C for 8h-night), as described in [41]. Seven-day old seedlings were inoculated with a solution of 10^3^ zoospores per plant, produced as described by Moussart et al. [54]. Disease severity was assessed on each plant seven days after inoculation, using a 0 (no symptoms) to 5 (dead plant) scoring scale proportional to the percentage of browning symptoms on roots and epicotyls [41]. A CC root rot index (CC_RRI) was calculated as the mean disease score on all plants in a pot.

In the field, the collection was evaluated for Aphanomyces resistance in infested nurseries over four years (from 2010 to 2013) and three locations in France (Riec-sur-Belon, Finistère (RI); Dijon-Epoisses, Côtes d’Or (DI) described in [41]) and in the USA (Kendrick, Idaho (KEN)). The collection was phenotyped each year at the two French locations and in 2012 at KEN, USA, for a total of nine environments. Field assays were carried out using randomised complete block designs with three replicates and an adjacent susceptible control (Solara) every two lines, as described in [41]. Two disease criteria were used to assess resistance in each plot, as reported in [41]: (i) a field root rot index (Field_RRI), using the 0–5 CC scoring scale, evaluated on ten plants per plot for each year in French nurseries and (ii) a field aerial decline index (Field_ADI), measuring yellowing symptoms on a plot, evaluated once or twice (Field_ADI1 and Field_ADI2) in all the USA and French disease nurseries, using a 1 (green plant) to 5 or 8 (dead plant) disease scoring scale depending on the nursery. The RRI and ADI scores on each plot were adjusted for local disease variation measured by scores on the adjacent susceptible control, as described in [41].

In the field, the collection was also phenotyped for developmental traits in a healthy nursery at Rennes-Le Rheu (Ille-et-vilaine, France (REN)) for two years (2010 and 2012), using a randomised complete block design with three replicates, as described in [43]. Earliness was scored on each plot as the number of calendar days to 50 % bloom (FLO1), to 100 % bloom (FLO2) and to ripening defined as 100 % of dried plants (RIPE) from the first day of the year. Average plant height (HT) was evaluated on five plants per plot at 100 % bloom.

### Genotyping

The collection was genotyped using three morphological genes (*Af*, afila/normal leaves; *R*, smooth/wrinkled seeds; *A*, anthocyanin production related to white/coloured flowers), specific-PCR markers for two known-function genes (Clpser and SugTrans, [55]), 45 simple sequence repeat (SSR) markers from [56] and 13,204 SNP markers from [49]. DNA was extracted from each pea line from approximately 1 g of young leaves collected on plants grown in a greenhouse, as described by Doyle and Doyle [57]. Concentrations were adjusted to 10 ng/μl for SSR genotyping and to 50 ng/μl for SNP genotyping.

SSR fragments were amplified by polymerase chain reaction (PCR) and analysed using GeneMapper® software v.4.0 (Applied Biosystems®, USA), as described in [44], except for the 20 RILs of the collection which were genotyped for SSRs as in [41]. Out of the 45 SSRs, 28 and 17 were located within and outside the main genomic regions previously associated to Aphanomyces resistance, respectively (Additional file 2) [41, 43]. Each SSR revealed between two and 16 alleles in the collection. Each line of the collection was coded for each SSR allele as homozygous for the considered allele (“BB”), heterozygous (“AB”) or homozygous for another allele (“AA”). A total of 441 SSR coded alleles were included in the genotyping matrix.

The 13,204 SNPs were part of a large pea SNP resource of 248,617 robust filtered SNPs discovered from whole-genome Illumina HiSeq2000 sequencing of 16 diverse pea genotypes [49]. These 13,204 SNPs were all located in gene-context sequences, each originating from a separate transcript [58], and were included in the Illumina Infinium® BeadChip developed by Tayeh et al. [49]. Infinium genotyping and data analysis were conducted as described in Tayeh et al. [49]. A total of 12,067 SNPs were selected for clearly being bi-allelic in the collection. Each line was coded “AA” or “BB” when homozygous for the first or second allele and “AB” when heterozygous.

### Statistical analysis of phenotypic data

Phenotypic data obtained on the collection for resistance to *A. euteiches*, earliness and height were analysed, for each variable in each field environment or CC experiment (individual analysis), and then for all year x location field environments (global analysis), using the R 3.1.1 program [59]. In the individual analysis, phenotypic variables were analysed using a linear model (LM) [R function lm] including G (genotype) and *R* (replicate) as fixed factors. In the global analyses, E (environment) and GxE (genotype x environment) interaction were added as fixed factors. Normality of residuals and homogeneity of their variances were checked using Skewness, Kurtosis and Shapiro-Wilk statistics (P ≥ 0.05), as well as Bartlett test (P ≥ 0.05), respectively [R functions skewness and kurtosis of package agricolae, [60]; plotresid of package RVAideMemoire, [61]; bartlett.test]. Mean-based heritability (h^2^) was calculated for each variable from variance estimates in the individual and global LM analysis, using the formulas h^2^ *=* σ_G_^2^/[σ_G_^2^ + (σ_E_^2^/*r*)] and h^2^ *=* σ_G_^2^/[σ_G_^2^ + (σ_GE_^2^/*E*) + (σ_E_^2^/*rE*)], respectively, where σ_G_^2^ is the genetic variance, σ_GE_^2^ the GxE interaction variance, σ_e_^2^ the residual variance, *E* the number of environments and *r* the number of replicates per line. Least Square Means (LSMeans) were calculated from each LM analysis (R function lsmeans of package lsmeans [62]). Histograms of LSMeans frequency distributions were drawn using the R function hist.

Pearson correlation analysis was carried out between LSMeans obtained from the individual and global analysis (R function corr.test of package psych, [63]). The significance of the Pearson correlations was evaluated with a false discovery rate correction for multiple testing (corrected *p-value <* 0.05; [64]). The heatmap of the Pearson coefficients (*r*) was drawn using the R function heatmap.2 (package gplots) [65].

A Multiple factor analysis (MFA) was performed for the different resistance variable categories (CC_RRI; Field_RRI; Field_ADI1 and Field_ADI2), using LSMeans from individual and global LM analysis (R function MFA of package FactoMineR, [66]). For MFA, LSMeans missing values were handled with the R package missMDA [67].

### Genetic analysis

The genotyping dataset of the collection, composed of three morphological genes, two specific-PCR markers for known-function genes, 441 SSR alleles and 12,067 SNP markers was filtered using PLINK 1.9 software [68–70]. Six of the 175 pea lines with missing data for more than 10 % of SNP markers were not included in the GWA analysis. Markers with missing data that exceeded 10 % or with a minor allele frequency (MAF) that did not exceed 5 % in the 169 remaining lines, respectively, were also removed for the GWA analysis. A total of 9980 markers, including three morphological genes, two specific-PCR markers of known-function genes, 189 SSR alleles and 9786 SNP markers were thus retained in the genetic analysis.

#### Missing data imputation

The raw dataset of 9786 SNP markers was imputed using the R function knncatimputeLarge (package scrime, [71]). This function imputed missing values, which corresponded to 0.45 % of the total dataset, regarding the k nearest neighbour SNPs without missing values. Imputation parameters were tested with 10 replicates, using a subset of 5001 SNP of the dataset with no missing values. For each replicate, 0.45 % of missing values were randomly simulated. Parameters tested were one to 10 nearest neighbours and four different methods to determine distances between SNPs [72]. Distance calculation methods were based on corrected Pearson’s contingency coefficient, scaled Manhattan distance, simple matching coefficient or Cohen’s kappa. Imputed values of simulated missing data were then compared to real values. The lowest error rate mean (9.86 %) over the 10 replicates was found with the Cohen’s kappa distance calculation method and six nearest neighbours. These parameters were applied to the 9786-SNP-markers and 189-SSR-allele dataset to impute missing values.

#### Linkage disequilibrium analysis

Pairwise LD between markers was explored within LGs from imputed genotypic data using PLINK 1.9 software. Obtained square correlation coefficient (*r*^*2*^) values were then plotted against genetic distances (cM) according to the consensus map from Tayeh et al. [49], namely TMap in this study, to estimate the LD decay. LD decay regression curves were fitted to the observed LD decay plots (R 3.1.1 program; [59]), following Sved [73] method with *r*_expected_^2^ = 1/(1 + 4N_e_ × c), *N*_*e*_ effective population size and c recombination rate between two markers. A nonlinear model was fitted on the pairwise LD data, then least-squares estimates were computed (R function nls) and *Ne* was predicted from this fitted nonlinear model (R function predict). The LD decay rate of the population was measured as the genetic distance (cM) where the average *r*^*2*^ dropped to half its maximum value (*r*^*2*^ *=* 0.5) [74].

#### Population structure and individual relatedness

A principal Component Analysis (PCA) and a Kinship relatedness matrix were used to estimate the structure of the collection from genotypic data, using the EMMA (efficient mixed-model association) method in the GAPIT (Genome Association and Prediction Integrated Tool) R package ([75], see the GAPIT R script at [76]). PCA and Kinship matrices were calculated based on a subset of 2937 SNP markers, among the 9980 imputed filtered markers on the TMap, corresponding to a single randomly-chosen marker per genetic position. Regarding to eigenvalues, the three first principal components (PCs) of the PCA were selected to take into account structure population in the GWA analysis. Clustering of individuals was considered based on their Kinship coordinates and was implemented in the GAPIT package with the UPGMA (Unweighted Pair Group Method with Arithmetic mean) method.

#### Association mapping

GWA analyses were performed using a modified version of the multi-locus mixed model (MLMM) R package [11]. Briefly, the PCA matrix of population structure and the Kinship matrix obtained from GAPIT were defined as cofactors in the MLMM (see the mlmm_cof.r R script at [77]). Significant SNP markers were also used as cofactors in a forward/backward approach.

The initial script of mlmm_cof was modified to define the multiple-Bonferroni (mBonf) threshold using the formula: mBonf = [−log(α/m)], with α *=* 0.10, the overall false positive threshold and m *=* 2937, the number of markers selected at non-redundant genetic positions on the TMap. Thus, the mBonf threshold was set at 4.47, which corresponded to a *p-value* of 2.5E-5. GWA study was performed from LSMeans scores of all the variables described in the phenotypic data analyses section, as well as from coordinates of lines on the two first PCs of MFA resistance variables analysis namely MFA.Dim.1 and MFA.Dim.2. In each GWA analysis, the optimal MLMM step was determined as the largest stepwise mixed model regression in which all cofactors have –log (*p-value*) above the mBonf threshold defined. At the optimal MLMM step, reliability of the analysis was evaluated based on plot of mBonf criteria among forward and backward steps, qqplot at the optimal step with mBonf criteria, and plot of partition of variance among steps (Additional file 3). The MLMM outputs used in this study were the partition of variance (percentage of variance explained by PCA, Kinship, markers in cofactors, and unexplained variance), the number and names of markers as cofactors at the optimal step and the *p-value* and allelic effect of each significant marker.

Local LD analysis was used to define the CIs around significant associated markers detected by GWA study using Plink 1.9 software. A LD block was determined as the interval containing all markers in LD (*r*^*2*^ *>* 0.2) with the significant associated marker, as described in [17]. LD blocks were named with the LG name (Roman numeral) and then an Arabic numeral in genetic position order. A LD block was considered as consistent for each trait (resistance to *A. euteiches*, or earliness or height), when it contained either one marker associated with at least two variables of the trait, or two or more linked markers (*r*^*2*^ *>* 0.2), each associated with at least one variable of the trait.

#### Haplotype analysis

At each consistent LD block associated with Aphanomyces resistance, marker haplotypes, *i.e.,* genotypic patterns at the given LD block, were identified among all the lines of the collection based on non-imputed raw genotyping data. Haplotypes were named with the LD block name and an Arabic numeral. For each trait significantly associated with marker(s) in a given consistent LD block, mean phenotypic scores of pea line groups comprising more than eight lines (5 % of the total number of lines) and carrying different haplotypes were compared, using the Tuckey-HSD test (α *=* 5 %; R package multcomp, [78]). Favourable and unfavourable haplotypes were defined as those meeting the three following criteria: (i) carrying favourable and unfavourable allele(s), respectively, at the disease trait-associated marker(s), (ii) without missing or heterozygous genotypic data at the other markers in the LD block and (iii) showing a significantly lower or higher disease mean score (breaking of group means considered, *P <* 0.05) than the other favourable or unfavourable haplotypes, respectively, for the highest number of disease traits among those associated with the LD block. According to these criteria, more than one favourable or unfavourable haplotype per LD block could be defined. Missing haplotypes were defined in lines showing missing genotypic data or heterozygosity for at least one marker in the considered LD block. Each line of the collection was described for its number of favourable haplotypes at all the consistent LD blocks. A Tukey-HSD test (α *=* 5 %) was then used to compare the mean numbers of favourable haplotypes in three groups of pea lines without missing haplotypes. The three groups were defined based on their MFA.Dim1 scores and corresponded to lines with high (25 % lowest scores), intermediate (50 % interquartile scores) or low (25 % highest scores) levels of resistance, respectively.

#### Comparative mapping

The consensus map from Hamon et al. [43], namely HMap in this study, summarized individual- and meta-QTL previously mapped for Aphanomyces resistance and developmental traits [41, 43], while the consensus TMap of Tayeh et al. [49] contained all the markers used in the present study. Thus, for comparative mapping, markers and QTL of the HMap were projected onto the TMap, using Biomercator V4.2 software [79]. The level of connectivity between the two maps was estimated using the “InfoMap” tool of the software. Maps were compiled using the “iterative map projection” tool from Biomercator V4.2 based on loci position data. TMap was used as reference map and HMap was projected based on common loci. Inversions of common loci were automatically resolved. Visualization of loci detected by association and linkage mapping on the resulted consensus map (namely THMap) was computed using MapChart 2.1 software [80].

#### Identification of putative candidate genes

Using annotation data for SNP anchored sequences from [49], a search was carried out for the putative gene and protein functions contained in each LD block for resistance to *A. euteiches*, earliness and height. The annotation data described predicted protein functions for each transcript sequence. The annotation was obtained following Blastx searches against *P. sativum*, *M. truncatula*, *G. max* and *A. thaliana* protein sequences. If at least two annotations were consistent, others missing, and at least one e-value was lower than 1E-50, the corresponding putative protein function was assigned to the sequence underlying the SNP. The putative annotation of the SNP-anchored sequence was scored with a ‘disagreeing hits’ comment when annotations disagreed, and with a ‘not assigned’ comment when at least three annotations were missing and/or all e-values were higher than 1E-50.. When possible, putative protein functions were attributed to known mechanisms of plant development and responses to biotic stress, based on the literature. SNPs which were anchored to previously cloned pea genes and were located close to LD blocks associated with resistance or developmental variables were identified based on information provided in [49].

## Results

### Analysis of phenotypic data

#### Resistance to A. euteiches

Global statistical analysis of RRI and ADI disease scores, obtained on the pea-Aphanomyces collection in field infested nurseries over the nine environments studied, showed highly significant GxE interactions (*P <* 0.001) (Additional file 4). These results confirmed the relevance of data analysis in each environment. Individual analysis of disease scores in each field environment, as well as in each CC experiment, revealed highly significant genotypic effects (*P <* 0.001) for all the disease variables but for Field_RRI at RI in 2013 (*P <* 0.01) and Field_ADI2 at RI in 2010 (*P <* 0.05) (Additional file 4). Heritability of resistance ranged from 0.28 (Field_RRI, RI 2013) to 0.96 (Field_ADI2, DI 2012), depending on the variable, and was high for most of the resistance variables (h^2^ > 0.60, except for Field_RRI at RI in 2013 and Field_ADI at RI in 2010, 2011, 2013 and KEN in 2012). Heritability values were especially high for CC_RRI scorings (h^2^ *>* 0.78), especially with the Ae109 strain (h^2^ *=* 0.91). Frequency distributions of LSMeans values for each individual resistance variable tended to fit normal curves (Additional file 5), except for CC_RRI_09_Ae109 which showed a skewed distribution with some highly resistant lines.

#### Earliness and height

Global statistical analysis of earliness and height scores, obtained on the collection in the field healthy nursery at REN over two years (2010, 2012), showed highly significant GxE interactions (*P <* 0.001) (Additional file 4). Individual analysis of the scores in each year revealed a highly significant genotypic effect (*P <* 0.001). Heritabilities of earliness and height traits in each environment were very high (h^2^ *>* 0.87). Frequency distributions of LSMeans values for each individual developmental variable tended to fit normal curves and were consistent between the two years (Additional file 5).

#### Correlations among variables

All Field_RRI and CC_RRI scoring data were significantly and positively correlated between each other (corrected *P <* 0.001, 0.30 *< r <* 0.79), as well as closely clustered, except that obtained with the Ae109 strain (Additional file 6). Most of the Field_ADI data were slightly and positively correlated to CC_RRI data (corrected *P <* 0.01, 0.25 *< r <* 0.57) with the RB84 strain but not with the Ae109 strain. Most of the Field_ADI1 and Field_ADI2 scoring data (68 %) were also significantly and positively correlated between each other (corrected *P <* 0.01, 0.22 *< r <* 0.79). Field_ADI scoring data were significantly and positively correlated with Field_RRI scoring data (corrected *P <* 0.03, 0.19 *< r <* 0.79), except those from 2011 (RI and DI).

All earliness and height data were significantly and positively correlated between each other (corrected *P <* 0.005, 0.26 *< r <* 0.92) (Additional file 6). Earliness data were slightly and negatively correlated with most of the Field_ADI data (corrected *P <* 0.05, −0.5 *< r < −*0.2).

#### Multiple factors analysis

**Fig. 1:**
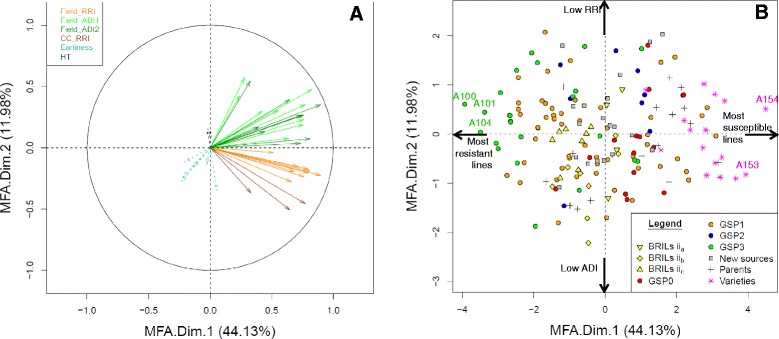
Graphical representation of Multiple Factors Analysis (MFA) of the phenotypic data. Correlation circle of variables (coloured according to groups) (**a**) and genotype factor map (coloured according to origin) (**b**), on the first two principal components of the MFA of disease variables. Earliness and height variables are supplemental variables and thus are projected onto the dimensions but were not included in the analysis. All the variables are abbreviated as described in Table 1. GSP0-3: lines from the GSP breeding program containing in their pedigree zero to three of the previously described major sources of resistance (*PI180693, 90–213* and *552*) [43] (Additional file 1), respectively. Parents: Parental lines of GSP breeding programs and/or RIL populations. BRILs ii_a_, ii_b_, ii_c_: Selected lines from the *Baccara* x *PI180693, Baccara* x *552* and *DSP* x *90*–*2131* RIL population, respectively; New sources: Selected accessions from the large INRA screening program; Varieties: Registered dry pea varieties. Extreme genotypes are labelled on the two main principal components

The two first PCs of the MFA analysis of disease resistance variables explained a total of 56 % of the inertia (MFA.Dim.1: 44.13 % and MFA.Dim.2: 11.89 %; Fig. 1a). Three groups of variables could be distinguished, including CC_RRI, Field_RRI and Field_ADI. A total of 77 % of the variables, especially the CC_RRI_RB84 variables, were highly correlated with MFA.Dim.1 (*r*^*2*^ *>* 0.5) and well represented on the first axis (cos^2^ *>* 0.5) (Additional file 7).

Pea lines with extreme negative or positive coordinates on MFA.Dim.1 were considered as the most resistant or susceptible lines, respectively. MFA.Dim.2 was mainly constructed from RB84 CC_RRI variables (*r*^*2*^ ≤ −0.5) in contrast to Field_ADI variables (*r*^*2*^ *>* 0.5). Pea lines with extreme negative or positive coordinates on MFA.Dim.2 had low levels of aerial symptoms or root symptoms, respectively. Dispersion of the pea lines on the two principal MFA components showed that the frequency of resistant and susceptible lines was homogeneous among the collection and within some groups of lines (Fig. 1b), which is optimal for GWA analysis of the resistance. The three most resistant lines were GSP breeding lines, namely *AeD99OSW −45-8-7* (*A100*), *AeD99OSW −50-2-5* (*A104*) and *AeD99OSW −47-6-1* (*A101*). The two most susceptible lines were *Safranos* (*A154*) and *Marignan* (*A153*), registered as French varieties.

### Genetic analyses

#### Linkage disequilibrium, structure, and kinship in the pea-Aphanomyces collection

From the imputed genotypic data of the collection obtained with the filtered 9980 markers, the LD decay was estimated to range from 0.08 to 0.16 cM, depending on the LG, and averaged 0.12 cM over all the pea LGs (Additional file 8). Based on non-redundant marker positions on the consensus genetic map of Tayeh et al. [49] (TMap), the average distance between two markers used in the analysis was 0.27 cM.

From the defined subset of 2937 SNPs located at non-redundant genetic positions on the TMap, PCA analysis of the collection identified three first PCs that explained a total of 20.91 % of genetic variation in the collection (Additional file 9 D). The first PC contributed to 9.28 % of the variation and the second and third PCs to 6.34 and 5.29 %, respectively. Pea lines were clustered slightly on PCs 1 to 3 depending on their origin, mainly varieties, distinct RIL populations, and groups of GSP breeding lines with any, two or three partially resistant parents in their pedigree (Additional file 9 A to C). The first three PCs were added to the GWA model as cofactors to take into account this slight population structure. From the subset of 2937 SNPs, the Kinship matrix of genetic similarities revealed a moderate relatedness among lines of the collection (0.5 *< r*^*2*^ *<* 0.65 for the major part of the lines), including sub-groups with higher relatedness (*r*^*2*^ *>* 0.8) according to pedigree, end use or sowing type (Additional file 10). Thus, the Kinship relatedness matrix was also added to the GWA model as a cofactor.

#### GWA markers and confidence intervals

##### Disease resistance

GWA analysis identified a total of 79 markers, located on the seven LGs, associated with 33 global or individual Aphanomyces resistance variables, including (i) 28 Field_RRI or _ADI variables from nine field environments, (ii) the three CC_RRI variables and (iii) the two MFA variables (Table 1 and Additional file 11). Zero to nine markers were significantly associated with each variable, with a *p-value* that ranged from 8.82E-28 to 3.32E-05, depending on the marker. Zero to nine cofactors were thus retained in the MLMM for each disease resistance variable, explaining a total of zero to 68 % of the phenotypic variation depending on the variable and in accordance with the heritability of the trait. Missing heritability (unexplained variance) ranged from 6 to 85 % and PCA and Kinship individually captured between zero and 78 % of the variance, depending on the variable. Allelic effects of markers associated with Field_ADI variables fluctuated widely, depending on the marker (absolute values from 0.13 to 0.61) (Table 1 and Additional file 11). Markers associated with Field_RRI had similar allelic effects as those associated with CC_ RRI with the RB84 strain (absolute values from 0.13 to 0.29), but lower than those associated with CC_RRI with the Ae109 strain (absolute values from 0.27 to 0.81).

**Table 1:** Genome-wide association analysis results using the multi-locus mixed model (MLMM) method in the pea-Aphanomyces collection

Variable (a)	Number of markers (b)	Range of *p-value* (c)	Range of allelic effect (d)	% of phenotypic variance explained by			Unexplained variance (h)
				PCA (e)	Kinship (f)	Markers (g)	
Field_RRI_All	2	7.86E-11–8.10E-06	0.13–0.17	48 %	20 %	19 %	14 %
Field_RRI_10_RI	2	1.81E-05–2.37E-05	0.16–0.19	13 %	28 %	25 %	34 %
Field_RRI_11_RI	2	9.92E-08–6.58E-06	0.23–0.26	42 %	9 %	20 %	29 %
Field_RRI_12_RI	5	3.14E-07–1.50E-05	0.17–0.29	20 %	18 %	39 %	23 %
Field_RRI_13_RI	0	–	–	26 %	25 %	0 %	48 %
Field_RRI_10_DI	1	5.78E-06	0.17	24 %	28 %	10 %	38 %
Field_RRI_11_DI	1	5.90E-07	0.29	54 %	25 %	9 %	12 %
Field_RRI_12_DI	3	4.76E-06–7.46E-06	0.15–0.17	36 %	14 %	23 %	28 %
Field_RRI_13_DI	3	6.00E-08–2.17E-05	0.16–0.26	28 %	13 %	23 %	37 %
CC_RRI_09_RB84	1	5.40E-12	0.28	43 %	8 %	19 %	30 %
CC_RRI_09_Ae109	2	8.82E-28–1.60E-06	0.27–0.81	8 %	10 %	68 %	13 %
CC_RRI_13_RB84	3	7.34E-11–3.89E-06	0.17–0.26	47 %	6 %	22 %	25 %
Field_ADI1_All	4	7.86E-11–2.43E-06	0.20–0.32	32 %	25 %	27 %	16 %
Field_ADI1_10_RI	4	3.12E-08–2.25E-05	0.26–0.40	10 %	4 %	39 %	48 %
Field_ADI1_11_RI	0	–	–	5 %	50 %	0 %	45 %
Field_ADI1_12_RI	3	3.99E-07–8.98E-06	0.45–0.54	23 %	50 %	15 %	13 %
Field_ADI1_13_RI	5	3.85E-08–3.32E-05	0.13–0.25	31 %	0.001 %	37 %	32 %
Field_ADI1_10_DI	0	–	–	37 %	45 %	0 %	18 %
Field_ADI1_11_DI	0	–	–	2 %	17 %	0 %	81 %
Field_ADI1_12_DI	7	1.13E-10–2.53E-05	0.21–0.61	29 %	21 %	39 %	12 %
Field_ADI1_13_DI	2	2.53E-10–1.16E-08	0.36–0.43	35 %	15 %	26 %	23 %
Field_ADI1_12_KEN	1	2.64E-05	0.18	32 %	25 %	8 %	34 %
Field_ADI2_All	3	1.15E-07–8.27E-06	0.22–0.38	39 %	47 %	8 %	6 %
Field_ADI2_10_RI	0	–	–	15 %	0.004 %	0 %	85 %
Field_ADI2_11_RI	2	7.77E-07–3.61E-06	0.33–0.43	0 %	43 %	23 %	34 %
Field_ADI2_12_RI	0	–	–	39 %	47 %	0 %	14 %
Field_ADI2_13_RI	0	–	–	39 %	45 %	0 %	17 %
Field_ADI2_10_DI	6	7.56E-08–1.27E-05	0.26–0.46	20 %	0.002 %	38 %	42 %
Field_ADI2_12_DI	0	–	–	24 %	50 %	0 %	26 %
Field_ADI2_13_DI	9	3.25E-12–2.34E-5	0.26–0.45	36 %	5 %	45 %	14 %
Field_ADI2_12_KEN	1	2.55E-05	0.34	9 %	48 %	11 %	32 %
MFA.Dim.1	4	1.51E-08–1.26E-05	0.42–0.56	56 %	15 %	21 %	8 %
MFA.Dim.2	2	1.01E-05–1.48E-05	0.36–0.48	9 %	78 %	1 %	13 %
FLO1_10_REN	5	1.24E-17–1.97E-05	1.82–5.86	9 %	17 %	64 %	10 %
FLO1_12_REN	7	6.29E-16–3.31E-06	1.55–12.80	8 %	23 %	63 %	6 %
FLO2_10_REN	8	8.50E-12–2.21E-06	0.91–2.88	5 %	0.001 %	71 %	24 %
FLO2_12_REN	3	2.68E-15–3.69E-08	2.47–5.49	5 %	19 %	52 %	24 %
RIPE_10_REN	7	5.02E-13–7.74E-06	0.94–2.88	0 %	3 %	72 %	25 %
RIPE_12_REN	2	2.69E-17–4.63E-06	2.15–3.39	0 %	25 %	49 %	26 %
HT_10_REN	3	3.76E-26–5.00E-06	6.42–30.65	0 %	37 %	53 %	10 %
HT_12_REN	2	1.62E-23–2.55E-05	8.10–34.61	0 %	23 %	76 %	1 %

(a) Variables are abbreviated as follows: *CC* Controlled conditions experiments, *Field* Infested field experiments, *RRI* Root rot index, *IDA1* First aerial decline index, *IDA2* Second aerial decline index, *FLO1* date of 50 % bloom, *FLO2* date of 100 % bloom, *RIPE* date of 100 % dried plants, *HT* height of plants, *09 to 13* year of field evaluation, *All* Global variables over field environments, *RI* Riec-sur-Belon, France, *DI* Dijon-Epoisses, France, *REN* Rennes-Le Rheu, France, *KEN* Kendrick (ID), *USA, RB84 and Ae109* two pure-cultured strains; (b) number of markers used as cofactors at the optimal step of the multi-locus mixed model (MLMM) analysis; (c) Range of *p-values* of the significant markers, significance threshold is *p-value <* 3.4E-05 as described in Methods section; (d) Range of allelic effects of the significant markers in absolute values; Percentage of phenotypic variance explained by: (e) the principal component analysis (PCA) matrix of the collection, (f) the Kinship relatedness matrix among lines of the collection, (g) all cofactor markers and (h) the unexplained variance qualified as “missing heritability”

**Fig. 2:**
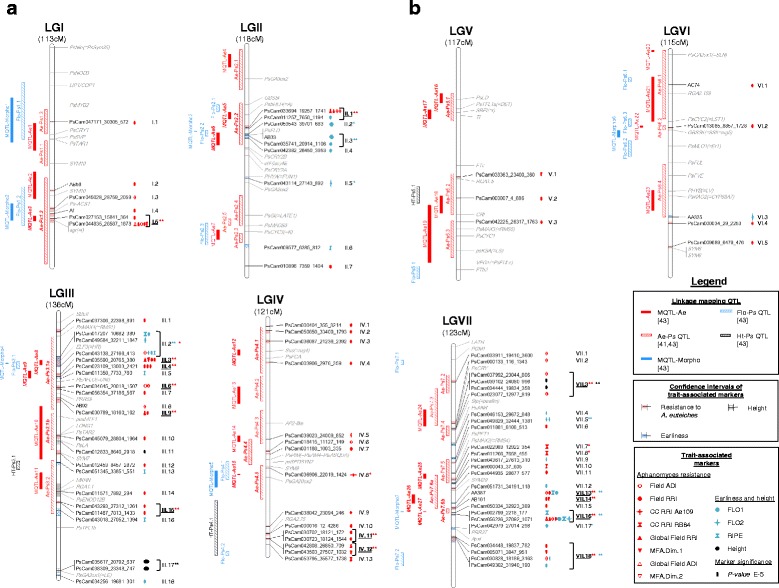
Comparative genetic map of genome-wide association (GWA) and previously detected linkage quantitative trait loci (QTL). The comparative genetic map was constructed from the projection of the consensus map from Hamon et al. [43] onto the consensus map from Tayeh et al. [49]. Linkage groups (LG) are named from I to VII and their size is indicated in cM Haldane. Resistance-, earliness- and height- associated markers, LD blocks and QTL are indicated in red, blue and black, respectively. To the right of each LG: Names of significant trait-associated markers identified by GWA study are indicated. The shading in the LG bar represents the confidence intervals around the significant trait-associated markers, based on linkage disequilibrium (LD) value of *r* ^*2*^ *>* 0.2. Symbols shown on the right of each marker and described in the legend indicate trait(s) to which the marker was significantly associated. Sign width is proportional to the significance level (*p-value*) of the marker-trait association. Names of LD blocks are indicated to the extreme right of each LG. The brackets indicate markers that were attributed to a same LD block (*r* ^*2*^ *>* 0.2). LD blocks labelled by double and single asterisks correspond to consistent (at least two significant markers with *P <* 5E-10) and highly significant but not consistent (one significant marker with *P <* 5E-10) blocks, respectively. Genomic positions of cloned pea genes are indicated in grey. The resistance genes cluster identified by Tayeh et al. [49] is represented by light grey shading on the bottom of LGIII. To the left of each LG: Projected QTL and Meta-QTL described in [41, 43] are represented with the same colour trait codes as for markers detected by GWA study. Solid bars represent Meta-QTLs [43], while hatched bars represent initial QTL before meta-analysis [41, 43]. The main Aphanomyces resistance QTL and Meta-QTL [43] names are in bold italic

A total of 52 CIs, ranging from 0 to 5.4 cM, were defined around all the significant disease-trait-associated markers, which included markers in LD (*r*^*2*^ *>* 0.2) with the targeted marker(s). Three kinds of LD blocks were identified, based on their significance and consistency (Fig. 2): (i) Fourteen LD blocks were considered as consistent since these included two to six disease trait-associated markers (double red stars on Fig. 2; 3.25E-12 *< P <* 2.64E-5). (ii) Four disease LD blocks included a single highly significant disease trait-associated marker (single red stars on Fig. 2; 8.82E-28 *< P <* 1.13E-10). Three of these markers were associated with CC_RRI variables. (iii) Most of the disease LD blocks (67 %) included a single moderately significant disease-trait-associated marker (3E-08 *< P <* 3.3E-05).

Out of the 14 consistent LD blocks, seven were Field_ADI specific, three were Field_RRI specific and four were common to Field_ADI and _RRI. The seven markers associated with global Field_ADI variables were all located in common LD blocks as individual Field_ADI variables. Out of the two markers associated to the global Field_RRI variable, one was located in the same LD block as one containing individual Field_RRI variables. Even if CC_RRI and Field_RRI data were correlated (*r*^*2*^ *>* 0.5), no common LD block was detected. Among the four markers associated with MFA.Dim.1, two were included in Field_RRI specific LD blocks and one in a LD block that contained Field_ADI associated markers. The two markers associated with MFA.Dim.2 variable were included in Field_ADI specific LD blocks.

The THMap, which resulted from the projection of the HMap onto the TMap, was based on 144 common markers (13 to 29 markers per LG; Additional file 12), most of which were located in previous QTL regions. The projection led to a total of 41 inversions of marker positions, which did not likely affect the QTL projection.

Comparison of linkage and association mapping results revealed that 19 of the 27 genomic regions including individual *Ae-Ps* QTL and 12 of the 27 *MQTL-Ae* meta-QTL previously reported [41, 43] were also detected by association mapping (Fig. 2 and Additional file 11). (i) Eight out of the 14 consistent disease LD blocks (*I.5*, *II.1*, *III.3*, *III.4*, *III.6*, *III.9*, *VII.13* and *VII.14*) co-localized with four of the seven main *Ae-Ps* QTLs previously identified and used for NILs construction by Lavaud et al. [44], *i.e., Ae-Ps1.2*, *Ae-Ps2.2*, *Ae-Ps3.1a-b* and *Ae-Ps7.6a-b*. Moreover, the highly consistent LD block *VII.16* was located just 0.45 cM away from *Ae-Ps7.6b*. Two other consistent LD blocks co-localized with two less consistent *Ae-Ps* QTL (*Ae-Ps3.2* and *Ae-Ps7.3*). (ii) One of the four disease LD blocks including single highly significant disease-trait-associated markers (*IV.8*) co-localized with the main previously detected QTL *Ae-Ps4.5*. (iii) Among the 40 remaining disease LD blocks identified in this study, three co-localized with the previous main QTL *Ae-Ps4.1* and 11 were located in regions not previously reported for resistance to *A. euteiches*. The main QTL *Ae-Ps5.1* previously identified on LGV was not detected in this study.

##### Linkage between resistance and morphological or developmental traits

A total of 25 markers, distributed over four LGs, were significantly associated (1.24E-17 *< P <* 1.97E-05) with the six earliness variables scored in the pea-Aphanomyces collection in the REN healthy field nursery over two years (Table 1 and Additional file 11). Twenty LD blocks were defined around earliness-associated markers, covering 0.3 to 5.4 cM. Six were consistent, since they were associated with two to six variables, and had different allelic effects ranging from one to 8.4 days (double blue stars on Fig. 2; 1.24E-17 *< P <* 1.97E-05). Three LD blocks were not consistent but included highly significant single trait-associated markers and had high allelic effects from 2.5 to 12.8 days (single blue stars on Fig. 2; 6.29E-16 *< P <* 4.30E-10). Three of the 14 QTL previously identified in Hamon et al. (2013) for earliness, *i.e., Flo-Ps2.2*, *Flo-Ps2.3* and *Flo-Ps3.1*, were also detected by association mapping.

A total of five markers, corresponding to three LD blocks distributed over two LGs, were significantly associated with the two height variables scored (3.76E-26 *< P <* 2.55E-05). Two LD blocks (*III.7* and *VII.3*) on LGIII and LGVII, ranging from 0.5 to 2.4 cM, were consistent, since they each included two height-associated markers. In particular, LD block *III.17* was highly significant (3.76E-26 *< P <* 1.62E-23) and presented an allelic effect which contributed to height differences of more than 30 cm. None of the three LD blocks co-localized with previous QTL detected for height in [43] (*HT-Ps)*.

Out of the total 75 LD blocks identified for resistance to *A. euteiches*, earliness and height, only five (*II.1*, *III.2*, *VII.13*, *VII.16* and *VII.18*) were detected for both resistance and earliness, and one (*VII.3*) for both resistance and height (Fig. 2 and Additional file 11). In the five resistance and earliness common LD blocks, three SNP markers and one SSR marker (*AA387*) were associated with both resistance and earliness. Allelic effects at these markers were opposing for resistance and earliness, *i.e.,* the resistance-enhancing alleles conferred later bloom.

One LD block identified for resistance to *A. euteiches* also co-localized with the *Af* morphological gene which controls leaf type on LGI. No LD block co-localized with the *A* morphological gene (*i.e., PsbHLH* gene) which control anthocyanin production on LGII. Linkage between Aphanomyces resistance and normal leaves at the *Ae-Ps1.2* QTL reported in [41, 43] was thus confirmed, while linkage between resistance and coloured flowers at the *Ae-Ps2.2* QTL was broken.

#### Marker haplotypes

**Fig. 3:**
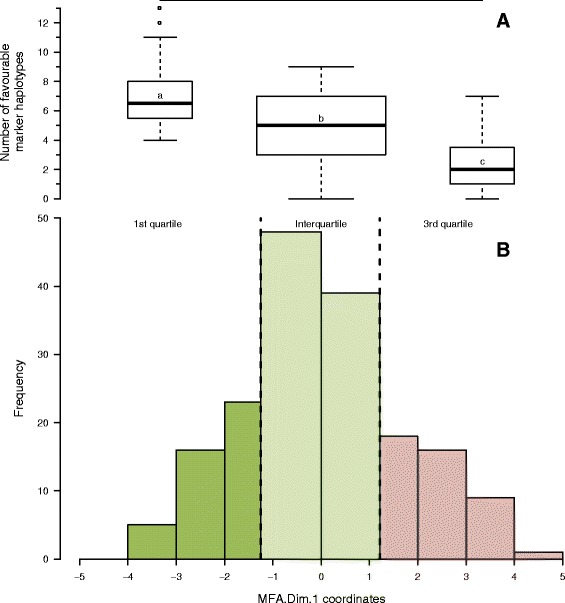
Mean comparison of favourable haplotype numbers in resistant, intermediate and susceptible lines. **a**: Box plot of the number of favourable linkage disequilibrium (LD) block haplotypes carried by pea lines (without missing haplotypes) from the pea-Aphanomyces collection belonging to the first quartile (25 % lowest values), interquartile (50 % intermediate values) and third quartile (25 % highest values) of coordinates on the first principal component of the multiple factors analysis (MFA.Dim.1). The three groups include pea lines with a high (first quartile), intermediate (interquartile) and low (third quartile) level of resistance. Letters indicate significantly different groups based on mean comparison (Tuckey-HSD, α *=* 0.05). **b**: Distribution histogram of MFA.Dim.1 for the pea lines (without missing haplotypes) of the collection. The first quartile (25 % lowest coordinates; dark green) carries the highest number of favourable haplotypes, the interquartile (intermediate values; light green) carries an intermediate number of favourable haplotypes and the third quartile (25 % highest values; light red) carries the lowest number of favourable haplotypes

At each of the 14 consistent disease LD blocks, three to 26 haplotypes were identified, depending on the LD block (Additional file 13). Mean comparison of phenotypic LSMeans between marker haplotype groups of a LD block, allowed one or two favourable haplotypes per LD block to be selected, except at LD block *IV.12* for which four favourable haplotypes were identified. A total of 22 favourable haplotypes were identified over the 14 consistent disease resistance LD blocks. A total of 112 haplotypes, carrying at least one favourable allele at the disease-trait-associated marker(s) of the blocks, were also identified among the 14 consistent disease resistance LD blocks. In a subset of 84 lines without missing haplotypes, the 25 % most resistant lines, according to MFA.Dim.1 coordinates, showed a mean frequency of favourable haplotypes which was significantly higher (eight favourable haplotypes on average for the 14 LD blocks) than that of the lines classified in the intermediate or susceptible groups (six and four favourable haplotypes on average, respectively) (Fig. 3 and Additional file 14). Lines *AeD99OSW-49-5-7* (*A103*), *AeD99OSW-45-8-7* (*A100*) and *AeD99OSW-37-3-4* (*A092*) showed the highest number of favourable haplotypes defined (13, 12 and 11 respectively) at the 14 consistent disease LD blocks in the subset of 84 lines. The group of susceptible lines carried the highest number of unfavourable haplotypes (three unfavourable haplotypes on average, versus two and one for intermediate and resistant groups, respectively). Two of the favourable haplotypes for resistance to *A. euteiches* (*II.1.02* and *VII.16.07*) were the worst haplotypes for earliness as it gave the latest flowering time. Another one (*II.1.06*) was unfavourable for earliness as it gave intermediate flowering time. One of the favourable haplotypes for resistance to *A. euteiches* (*VII.3.04*) was unfavourable for plant height as it gave higher plants. Early bloom and small height are two important selection criteria in dry pea breeding programs.

#### Putative candidate genes

Out of the 550 markers in the 52 disease-related LD blocks, 211 were located in gene sequences and could be assigned to putative protein functions (Additional file 15). Out of these 211 annotated SNPs, 135 SNPs corresponded to genes with putative functions reported to be involved in plant response to biotic stresses. Among these genes, 21 encode for protein domains reported as involved in plant recognition of pathogens (including Leucine Rich Repeat-LRR-domains), 65 for domains reported to contribute to signal transduction (including Serine-Threonine-Tyrosine Protein Kinases and proteins involved in Ethylene biosynthesis), 12 for proteins playing a role in transcription regulation (including a WRKY transcription factor, a VQ motif and a basic helix-loop-helix domain) and 47 for proteins involved in plant defence responses such as cell wall modifications (including homeobox leucine zipper proteins), oxidative burst (including Ras GTPases), detoxification (including an ABC transporter C and cytochrome P450 enzymes) or cell death (including chaperone Dnaj proteins). A subset of 24 putative proteins were also reported to be involved in plant growth or development, out of which 14 were not reported to be related to pathogen resistance. Four disease-trait-associated markers in LD blocks *III.14*, *IV.10*, *V.1* and *VI.1*, were close to (0.1–4.4 cM) but not in LD with pea resistance genes analogues (RGA) coding for nucleotide binding site Leucine Rich Repeat (NBS-LRR) proteins (*RGA1.1*, *RGA2.75*, *RGA1.5* and *RGA2.159*, respectively) [81, 82]. Three RRI-associated markers in LD blocks *III.12*, *IV.8* and *VII.11*, and one Field_ADI-associated marker in LD block *VI.5*, were close (0.5–0.8 cM) to cloned genes involved in pea-rhizobia symbiosis (*SYM7*, *SYM9*, *SYM29* and *SYM8*, respectively). A Field_ADI- associated marker in LD block *I.5* co-localized with the *SGR* gene (*Stay Green*) which controls chlorophyll catabolism during foliage senescence [83]. Among the 18 consistent or significant disease-related LD blocks, 56 SNP markers were attributed to putative sequences coding for interesting candidate proteins involved in plant resistance to pathogens, including Leucine-rich repeats (LRR) in LD blocks *I.5*, *IV.8* and *VII.3*, Serine/threonine protein kinases in six LD blocks [84] and an ABC transporter [85] in LD block *III.6.*

Among the 15 additional LD blocks exclusively related to earliness, 55 of the 110 SNP markers could be assigned to a putative function, 24 of which are involved in plant growth or development. Three earliness-associated markers in LD blocks *II.3*, *III.2* and *III.5*, were close (0–2.1 cM) to cloned genes involved in flowering time or indeterminacy (*PsFLD*, *Hr* and *PEAFLO* respectively) [86–88]. Among the three height-related LD blocks, 26 of the 44 SNP markers could be assigned to a function. Eight of them were reported to be involved in plant growth and development, including two SNPs in LD blocks *III.11* and *III.17* designed in sequences of pea cloned genes controlling plant height, *i.e., La* (*PsLA*) [89] and *Le* (*PsGA3ox1*) [90], respectively. The height-associated markers in the third height-related LD block *VII.3* was located just 0.8 cM from the SNP marker designed in the sequence of the *Cry* gene, involved in plant growth [89].

## Discussion

This work describes the first GWA approach in pea to decipher polygenic control of partial resistance to *A. euteiches,* using novel plant material as well as high density and quality genomic resources. The study evaluated a pea-Aphanomyces collection of 175 lines, enriched in sources of resistance to *A. euteiches*, which were identified in the last 15-years of research and breeding programs. Genetic diversity and recombination events screened in the collection were higher than those previously studied in bi-parental populations. We took advantage of the GenoPea Infinium ® BeadChip recently generated [49], containing 13,204 SNPs precisely anchored onto a consensus genetic map generated from genotyping data on more than 1300 RILs. This novel genomic resource greatly enhanced the resolution of the genetic analysis in the association mapping collection compared to the few hundred genetic markers used in previous linkage studies of Aphanomyces resistance in pea [41, 43]. We used the same resistance phenotyping methodologies and Aphanomyces field network as in the previous QTL analysis of resistance, which led to high accuracy when comparing the results of these two approaches. Finally, this study used a MLMM to perform GWA study. This model corrects for population structure and individual relatedness, and takes into account significantly associated SNPs as cofactors to detect other SNPs. This approach limits the number of false positive and false negative associations [11].

Specifically, the GWA study (i) identified 14 consistent loci out of a total of 52 small sized LD-based CIs detected for resistance to *A. euteiches*; (ii) validated and refined six of the seven major resistance QTL previously identified; (iii) confirmed or broke negative associations with morphological and developmental loci; (vi) pinpointed interesting haplotypes and determined their frequency in the most resistance pea lines, for application in breeding; (v) identified relevant and interesting putative candidate genes underlying main resistance loci.

### GWA study validated most of the previously detected QTL and identified new resistance loci

This study validated most of the previously detected Aphanomyces resistance QTL in pea. Out of the 52 resistance-associated loci, 41 were estimated to be located in 19 of the 27 projected *Ae-Ps* QTL, including six of the seven main consistent QTL, and 12 of the 26 projected meta-QTL (*MQTL-Ae)* previously identified from bi-parental population studies [41, 43]. Four and two of the main previously consistent resistance QTL (*Ae-Ps1.2*, *Ae-Ps2.2*, *Ae-Ps3.1*, *Ae-Ps7.6a-b* and *Ae-Ps4.1, Ae-Ps4.4-4.5)* were re-detected in this study by eight of the 14 consistent resistance loci and seven of the other single-variable specific loci, respectively. Twelve previously detected minor-effect QTL were also confirmed with 18 resistance-associated loci, including two that were consistently detected. GWA validation of previous linkage resistance QTL was expected, as the pea-Aphanomyces collection was enriched in genetic pools derived from sources of resistance studied in previous QTL analysis (*90*–*2131*, *552* and *PI180693*). A total of 87 % of the 121 GSP breeding lines and RILs of the collection were derived from at least one of these three sources of resistance. In crops, GWA studies usually used panels with a good representation of species genetic diversity, such as diversity collections developed in pea [52, 91]. For pea Aphanomyces resistance, the GWA study required an adapted panel with a higher resistance allele frequency than to the one previously observed in the pea natural genetic variation (Pilet-Nayel et al., unpublished) [36]. However, Kwon et al. [50] identified a SSR marker (PSARGDECA_F) associated to Aphanomyces resistance [34] in the USDA pea core-collection, which is estimated to be located in the *Ae-Ps7.6* QTL region, close to LD block *VII.11*, using comparative genetic maps [43, 45, 49]. Marker and methodology tools were also used to optimize comparative analysis of GWA and previous linkage results. Most of the SSR markers, previously associated with resistance QTL [43], were integrated into the consensus SNP genetic map of Tayeh et al. [49]. Common markers were used as bridges for comparative mapping through linkage QTL projection. Furthermore, the field and climate chamber conditions were the same in [41, 43] and in this study, which increased the accuracy of comparative mapping.

Eight of the 27 projected *Ae-Ps* QTL previously associated with the resistance [43] were not identified by GWA in this study, including one main consistent QTL (*Ae-Ps5.1*). Several factors could lead to non-validation of QTL by GWA. These include the low detection power of minor-effect QTL, low allele frequency, GxE interactions, epistatic interactions and gaps in marker coverage [5]. In this study, the detection power of low effect QTL may have not been optimal due to the moderate population size (175 lines). Markers with low MAF (MAF *<* 5 %) were sometimes clustered in some genomic regions, such as on LGV, and were removed from the analysis, creating gaps in the genome-wide scan of marker-trait associations. GxE interactions for field Aphanomyces resistance were observed in this study over the nine environments tested, as previously reported and discussed regarding pedo-climatic conditions, pathogen diversity and the presence of other root rot pathogens [60]. They may have resulted in the detection of QTL specific to the field environments presently studied compared to previous ones, which were submitted to different climatic conditions and pathogen population pressures. Epistatic interactions have also been reported for Aphanomyces resistance [41] and some QTL effects were recently shown to increase or be lost when transferred into a new genetic background [44]. Finally, despite the unprecedented marker density used in GWA analysis in pea, marker coverage gaps cannot be excluded, due to the restricted choice of SNPs in the Infinium SNP chip used and the unavailable information on physical distances between markers. The non-detection of the main consistent QTL *Ae-Ps5.1* was particularly unexpected since it was identified from CC experiments with several strains including RB84 and Ae109 used in this study, and contributed up to 38 % of the phenotypic variation in the DSP x 90–2131 RIL population [43]. However, Lavaud et al. [44] recently showed that the effect of *Ae-Ps5.1* was lost when transferred into pea lines other than *DSP*, suggesting epistatic interactions between this QTL and genetic background. In addition, a very low allele frequency was observed for the SNP designed in the *Ae-Ps5.1* closely linked marker *PsLD* (MAF *=* 0.8 %) [41, 86] and for its neighbour SNP in high LD (*r*^*2*^ *=* 0.55; MAF *=* 1.4 %), which were both consequently removed from the analysis.

In this study 11 new Aphanomyces resistance loci were identified, including three consistent ones (*IV.11, IV.12* and *VII.18*). Comparative mapping was based on few markers (mainly SSRs) which resulted in some inversions. Thus, some projection inaccuracies may have wrongly pointed out these loci as new ones, especially for the *VII.16* interval. However, the higher resolution of GWA analysis may also have highlighted resistance alleles from the studied germplasm, which would not have been previously detected due to low marker coverage. Most probably, new favourable alleles are present in the pea-Aphanomyces collection, which included new sources of resistance [36] which were not investigated in previous linkage-QTL analyses.

In other pathosystems, GWA mapping often validated linkage mapping QTL and detected new loci. For example, Samayoa et al. [15] and Zhao et al. [14] reported similar results when comparing linkage and association mapping of Mediterranean corn borer resistance in maize and *Sclerotinia sclerotinium* resistance in soybean, respectively.

### GWA study detected resistance loci with a higher resolution than in previous linkage mapping studies

In the present GWA study, QTL resolution increased compared to previous linkage mapping studies of Aphanomyces resistance. The 52 resistance-associated intervals were detected with much smaller sizes (0–5.2 cM, depending on the locus, 0.9 cM in average) than those previously identified in QTL mapping studies (2.1–43.9 cM, depending on the *Ae-Ps* QTL, 17.4 cM in average). The LD decay rate in the pea-Aphanomyces collection was low (*r*^*2*^ < 0.5 within a map distance of 0.12 cM) compared to the one observed in a similar study in pea [9] (average *r*^*2*^ < 0.17 within a map distance of 5–10 cM). This low LD decay rate is particularly interesting as LD decay of autogamous (self-pollinated) species have generally been reported slow (~100kb), as in rice, foxtail millet or soybean, compared to outcrossing species (~2kb) [5]. This may be especially due to a high number of recombination events in the breeding lines coming from the GSP recurrent selection program (representing 58 % of the lines in the collection), which were mainly derived from double crosses between multiple parents (Additional file 1). The average distance between two adjacent marker positions of the TMap considered in this study (0.27 cM) was higher than the LD decay rate. Since marker genetic positions may not have covered all the putative recombination points in the collection, there is still the potential to increase genotyping density of the collection, to improve QTL detection resolution. Previous meta-QTL analysis of Aphanomyces resistance attempted to reduce QTL CIs but this reduction was potentially over-estimated because of the only partial independence on populations and environments compared [43]. The MLMM model recently proposed by Segura et al. [11], used cofactors that captured background genetic variance during genome scan to improve the precision of cofactor detection as found in Composite Interval Mapping.

Comparative analyses of the QTL detected by linkage and association studies are often not carried out on comparable genetic distance scales in both studies. Consequently, several association mapping loci could correspond to a single QTL interval identified by linkage mapping. Ingvarsson & Street [10] reported frequent splitting of linkage mapping intervals into much smaller association mapping intervals. Split association mapping intervals involved in a same larger linkage mapping interval could correspond to several linked genes controlling the trait or to a single imprecisely located region due to GxE effects on the phenotype. Our findings suggest linkage between multiple genes hypothesis when closely located LD blocks were detected from different variables, as seen for example for the *Ae-Ps7.6* region on LGVII (*VII.7-8* and *VII.13-16* blocks). In other cases the results were consistent with single genes hypothesis when neighbouring blocks were detected for the same type of variable, as in the *Ae-Ps3.1* region on LGIII (*III.3–4* for Field_RRI variables; *III.6–7* or *III.8–9* for Field_ADI variables).

### GWA study provides new tools for pyramiding Aphanomyces resistance alleles in breeding programs

This study identified marker haplotypes at consistent Aphanomyces resistance loci, the pyramiding of which was associated with increased levels of partial resistance in pea lines of the collection. At the 14 consistent LD blocks identified, 22 favourable haplotypes carrying the favourable alleles at disease trait-associated markers were significantly associated with enhanced resistance levels. The highest resistant lines of the pea-Aphanomyces collection carried a significantly higher number of favourable haplotypes. The five most resistant lines with no missing haplotypes were breeding lines from the AeD99OSW GSP program, all derived from direct crosses between the three most partially resistant germplasm studied in previous QTL analysis [(*90*–*2131* x *PI180693*) x *552*]. These five breeding lines had a higher level of partial resistance than their individual parents. They combined between eight and 13 of the favourable haplotypes selected at the 14 consistent resistance loci, whereas each of their single parental lines combined between three to nine favourable haplotypes. From this study, different combinations of haplotypes seemed to be related to enhanced levels of resistance. The best combinations were quite difficult to identify since they could vary depending on the lines. However, the favourable haplotypes defined at several blocks (*I.5*, *II.1*, *III.3*, *III.4*, *III.6* and *III.9*) appeared to be frequently represented in the most resistant lines.

Our findings suggested that the pyramiding of Aphanomyces resistance alleles with moderate to low effects is a powerful strategy to develop pea lines with increased levels of partial resistance to *A. euteiches*. In the past 30 years, such a pyramiding strategy, based on phenotypic recurrent selection schemes, was successfully developed for root rot diseases in public USA pea breeding programs [31, 32]. In particular, Lewis and Gritton [92] developed a phenotypic recurrent selection protocol consisting of one cycle per year, including selection of F_2_ lines on aerial symptoms of the plants in an infested field nursery, followed by intercrosses and selfing of the best lines. The *552* germplasm line was selected from the eighth cycle of recurrent selection (Roux-Duparque, Pers. Comm.), suggesting that this line accumulated resistance alleles. Accumulation of resistance alleles in the *552* germplasm line was demonstrated in this study (nine favourable haplotypes identified). More recently, Lavaud et al. [44] reported that the combination of two or three of the main consistent Aphanomyces resistance QTL identified in [43] could increase the partial resistance level, depending on genetic backgrounds. In several other pathosystems, combinations of resistance alleles were shown to successfully enhance resistance levels, including stem rust resistance in wheat [93, 94], spot leaf diseases resistance in spring wheat [95] and spot blotch resistance in barley [96].

We also identified negative linkages between resistance and morphological or developmental alleles, to be considered with caution for application in dry pea breeding. Undesirable linkages were previously reported between Aphanomyces resistance and coloured flowers, long internodes, normal leaves and late-flowering [43], and have been avoided in breeding programs [32]. In the present study, associations were highlighted between resistance and late flowering alleles at markers in the consistent disease LD blocks *VII.13*, *VII.16* and *VII.18*. In non-consistent disease LD blocks, undesirable links were also highlighted between resistance and normal leaves (LD block *I.4*) or late flowering (LD block *II.2*). In contrast, high resolution of association mapping in this study also enabled linkage between resistance and coloured flowers (*A* gene) alleles to be broken in the genomic region corresponding to the previous main resistance QTL *Ae-Ps2.1* (LD block *II.1*). Thus, different pea lines of the collection carrying favourable haplotypes in the LD block *II.1* have white flowers. However, in the most resistant lines of the collection, such as *AeD99OSW- 45-8-7,* some negative associations could not be broken, as those lines presented normal leaves and some of them still had coloured flowers. Due to the high resolution of GWA study, we could also hypothesize that loci controlling resistance and delayed leaf senescence (*SGR* gene) [83] in the LD block *I.5* are distinct since they were not in LD (*r*^*2*^ *>* 0.2). Morphological and developmental-trait-loci which are not associated with Aphanomyces resistance could also be detected, suggesting that resistance could be improved without including undesirable traits. For height and earliness, the height locus *III.17* on the *Le* gene and the earliness loci *II.2*, *II.3*, *II.5*, *VII.5* and *VII.17* were identified and not associated with resistance loci. Genetic associations between plant disease resistance and undesired developmental traits such as late-flowering have been commonly reported in the literature [2]. Association genetic studies could have confirmed such negative linkages [97] but sometimes allowed them to be broken, as observed in our study

### GWA study identified relevant putative candidate genes underlying Aphanomyces resistance QTL

The high resolution of the present association mapping study and the recent availability of pea transcriptome sequences connected to the 13,204 SNPs used in this study, constitute unprecedented advantages for identifying putative candidate genes underlying Aphanomyces resistance QTL. Indeed, the detection of plant height QTL in previously cloned genes involved in plant growth, and the proximity of flowering genes to earliness LD blocks suggests that the resolution of the present GWA study was high enough to pinpoint relevant candidate genes.

GWA study identified a high frequency (64 %) of putative candidate genes corresponding to a diversity of stress-related protein functions, underlying resistance-associated intervals. To date, the low number of resistance QTL cloned suggested that the molecular functions underlying them could be diverse [2, 3, 98]. Hypothetical gene-encoded proteins underlying resistance QTL included genes involved in plant-pathogen recognition, signal transduction, activation or repression of transcription, defence responses and developmental or morphological modifications with pleiotropic effects. Such a diverse range of genes underlying resistance intervals was also identified in this study. Putative candidate genes underlying major effect QTL *Ae-Ps4.4-4.5*, recently validated for pea resistance to the American *A. euteiches* strain Ae109 [44] (LD block *IV.8*), included basic helix-loop-helix (bHLH) and ethylene-responsive transcription factors and a NBS-LRR. A second main effect QTL *Ae-Ps7.6*, validated for pea resistance to the French *A. euteiches* strain RB84 (LD blocks *VII.8* and *VII.10*), included two putative infection-induced proteins. Some different types of putative stress-related genes clustered in the disease LD blocks identified in this study and thus may be strong candidates for underlying one of the known mechanisms. For example, LD blocks *III.9* and *I.5* contained six and four putative candidate genes, respectively, involved in mechanisms of pathogen recognition, transcription regulation, signal transduction and/or defence response.

Other LD blocks seemed to be interesting as they are very close to NBS-LRR cloned genes (*III.14*, *IV.10*, *V.I* and *VI.1*), however they were detected in single environments and had moderate effects. Genes encoding NBS-LRR protein domains are known to be clustered in crop genomes, such as described in *Medicago truncatula* [99], since they are known to be subjected to rapid evolution through local duplications. In pea, Tayeh et al. [49] recently reported a large cluster of 14 NBS-resistance-like genes at the bottom of LGIII. This genomic region, including SNP markers assigned to these 14 NBS-like genes, co-localized with disease LD block *III.14* and is close to consistent LD block *III.15* identified in this study.

Some disease LD blocks contained putative genes similar to those underlying disease resistance QTL cloned in plants, such as ABC transporters (*III.6*) and NBS-LRR proteins (*I.5*, *IV.8* and *VII.3*)). An ABC transporter protein have been shown to underlie the *Lr34* major QTL controlling durable resistance to leaf rust stripe rust and powdery mildew in wheat [100]. Other LD blocks could also be detected close to cloned symbiosis genes (*III.2*, *IV.8*, *VI.5* and *VII.11)*, suggesting possible common mechanisms between plant-pathogens and plant-beneficial organisms interactions [101]. Some LD blocks also included plant or root development genes (*IV.3*, *IV.7*, *VII.7* and *VII.10*), suggesting their involvement in disease resistance, tolerance or avoidance as reported for several pathosystems [100].

## Conclusion

In the present GWA study, most of the previously identified Aphanomyces resistance QTL were validated with a finer resolution than before, by taking advantage of the new plant and genomic resources developed in pea. New points of discussion were raised regarding comparison of association and linkage analysis to dissect polygenic disease resistances in plants. This study provides new tools for breeding, including pea germplasm and SNP markers associated with resistance, as well as useful information about marker haplotypes at main resistance loci and undesirable allele linkages with resistance. It demonstrates that pyramiding resistance alleles is a key strategy for increasing levels of partial resistance to Aphanomyces root rot in pea. The choice of resistance loci to be pyramided remains to be explored, especially using NILs [44]. This study also identified relevant candidate genes to be confirmed and validated in future studies. These will benefit from further genomic resources in progress, developed in part from a complete pea reference genome sequence becoming available, for increasing resolution of the GWA study.

### Availability of supporting data

Further data sets supporting the results of this article are included in Additional files 16 and 17.

## Additional files

Additional file 1:
**Description of the pea-Aphanomyces collection.** (a) Line categories, as referred in Fig. 1b and Additional file 9 in legends; (b) Line codes in the Biological resource center (CRB) database of INRA Dijon, France; (c) Country codes ISO3166-1; (d) Cv: Cultivar, Bl: breeding line, Gmp: germplasm, Lv: local variety, Wa: wild accession; (e) Fd: fodder, Gd: garden; (f) Ws: winter sown, Ss: spring sown; (g) Nl: normal leaves, af: afila leaves, (h) W: white, Pu: purple, Pi: pink; (i) Wr: wrinkled, Sm: smooth; #: parents of GSP breeding programs; $: parents of RIL populations. (XLSX 29 kb) Additional file 2:
**Simple sequence repeat (SSR) markers used to genotype the pea-Aphanomyces collection.** (a) main Aphanomyces resistance quantitative trait loci (QTL) defined in [43]. (XLSX 14 kb) Additional file 3:
**Example of MLMM R package plot outputs for analysis of the MFA.Dim.1 variable.** (A): Bonferroni correction of the highest marker *p-value* for each step of the forward and backward analysis. Optimal step (dashed blue vertical line) is determined as the largest stepwise mixed model regression in which all cofactors have –log_10_(*p-value*) above the mBonf threshold (dashed black horizontal line). (B): QQ-plot at the optimal step. (C): Partition of variance for each forward and backward step. Variance explained by population structure (PCA; grey); all markers used as cofactors (blue); Kinship matrix (green) and unexplained variance (*=* missing heritability; red). The dashed blue vertical line represents the optimal step according to mBonf threshold. (D) Genome-wide Manhattan plot without marker cofactors. The dotted line represents the multiple-Bonferroni threshold above which markers are considered as significant. (E): Genome-wide Manhattan plot at the optimal step with marker cofactors (4 red dots). The dotted line represents the multiple-Bonferroni threshold above which markers are considered as significant. (PDF 148 kb) Additional file 4:
**Statistical analysis results of Aphanomyces resistance, earliness and height scoring data in the pea-Aphanomyces collection.** (a) CC: controlled condition experiment, Field: Infested field experiment, RRI: Root rot index, ADI1: First aerial decline index, ADI2: Second aerial decline index, FLO1: date to 50 % bloom, FLO2: date to 100 % bloom, RIPE: date to 100 % dried plants, HT: height of plants, 09 to 13: year of experiment, All: global variables over all field environments, DI: Dijon-Epoisses, Burgundy, France, RI: Riec-sur-Belon, Brittany, France, REN: Rennes-Le Rheu, Brittany, France, KEN: Kendrick, ID, USA, ‘$’ Residual normality statistics are not exact but graphs of residuals are correct, (b) Mean, minimum, maximum and standard error in the pea-Aphanomyces collection; (c) ANOVA significance *p-value* codes: 0 *<* ‘***’ *<* 0.001≤ ‘**’ *<* 0.01≤‘*’ *<* 0.05≤ ‘ns’ *<* 1; (d): Genotype effect; (e): Replicate effect; (f): Environment effect; (g): Genotype x Environment interaction effect; (h) mean-based heritability. (XLSX 16 kb) Additional file 5:
**Frequency distribution of least square means obtained for Aphanomyces resistance, earliness and plant height in the pea-Aphanomyces collection.** Least square means were obtained from analysis of variance for three Aphanomyces resistance traits (Root Rot Index, first and second Aerial Decline Indexes, coded RRI, ADI1 and ADI2, respectively), three earliness traits (dates to 50 % bloom, 100 % bloom and 100 % dried plants, coded FLO1, FLO2 and RIPE, respectively) and plant height (HT). Aphanomyces resistance traits were assessed over nine infested field environments and against two reference strains of *A. euteiches* (RB84 and Ae109) in controlled conditions. Earliness and height were assessed in two healthy environments. n: total number of pea lines assessed; m: mean ± standard deviation of the pea-Aphanomyces collection; h^2^: mean-based heritability. (PDF 38 kb) Additional file 6:
**Clustered heatmap of correlation coefficients between disease resistance, earliness and height variables data.** Variables are coded as described in Additional file 4. Only correlations with a *p-value <* 0.05 are shown. Colours represent level of correlation, coded as follow: dark red: *r <* −0.8; medium red: −0.8 *< r <* −0.5; light red: −0.5 *< r <* −0.2; white: −0.2 *< r <* 0.2 or *p-value >* 0.05; light green: 0.2 *< r <* 0.5; medium green: 0.5 *< r <* 0.8; dark green: *r >* 0.8. Clustering is based on the UPGMA method. (PDF 48 kb) Additional file 7:
**Variable contribution in the two first Multiple Factor Analysis (MFA) principal components.** (a) Variables are coded as described in Additional file 4, (b) percentage of variable contribution in the construction of the MFA axis, ‘*’: the six highest contributions; (c) cos^2^ indicates the representation quality of the variable on the MFA axis, ‘*’: 0.2 *<* cos^2^ *<* 0.5, ‘**’: cos^2^ *>* 0.5; (d) correlation between variable and MFA axis, ‘*’: 0.5 *< r* ^*2*^ *<* 0.8, ‘**’: *r* ^*2*^ *>* 0.8. (XLSX 17 kb) Additional file 8:
**Linkage disequilibrium (LD) decay in the pea-Aphanomyces collection.** Coloured curves represent the estimated LD decay for each linkage group (LG). Dashed horizontal lines represent half of the maximum LD value (LD threshold=0.5). Arrows represent the LD decay rate, as the estimated genetic distance (cM) to drop to the LD threshold on each LG. (PDF 157 kb) Additional file 9:
**Population structure of the pea-Aphanomyces collection based on Principal Component Analysis (PCA).** PCA from GAPIT R package [75] based on 2937 markers. Distributions of pea lines of the collection are represented on the first three principal components (A–C), which explain a total of 20.91 % of inertia (D). Categories of pea lines are described in Fig. 1 and Additional file 1. (D) Inertia contribution of each principal component (from PC1 to PC169). (PDF 151 kb) Additional file 10:
**Clustered heatmap of the Kinship matrix.** Kinship matrix from the GAPIT R package [75] based on 2937 SNP markers. Clustering is based on the UPGMA method. Colours represent the degree of relationship between two given lines. Pea line information is described as in Additional file 1. Lines sharing same pedigree or end use or sowing type are well clustered thus the Kinship matrix efficiently represents the relationships between individuals. (PDF 1253 kb) Additional file 11:
**Genetic loci associated with Aphanomyces resistance, earliness and plant height detected by genome-wide association mapping using the MLMM method.** (a) Genetic positions are estimated on the genetic map resulting from the projection of the marker map from Hamon et al. [43] onto the consensus map from Tayeh et al. [49]; (b) A linkage disequilibrium (LD) block is designed with all markers in LD (*r* ^*2*^ *>* 0.2) with the associated marker. LD blocks are named in consecutive numerical order following their linkage group (LG) name and their genetic position; (c) Aphanomyces resistance and earliness Meta-QTL (quantitative trait loci) described in [43]; (d) Initial QTL for Aphanomyces resistance and earliness before meta-analysis described in [41, 43]; (e) Trait-associated marker(s) and their genetic position(s) in the LD blocks. *PsCam*- markers are SNPs developed by Tayeh et al. [49], *Af* is a morphological gene involved in leaves shape, other markers are SSRs (detected allele in brackets); (f) Minor allele frequency (MAF) of each detected marker in the collection; (g) Variables are coded as described in Additional file 4; (h) significant *p-value* of the marker-trait association *<* 2.5E-5; (i) mean value of the trait corrected with the effect of all markers included in the MLMM analysis; (j) allelic effect of the marker on the variable, the sign depends on the allele code; (k) standard error at the marker around the mean value of the trait in the collection. (XLSX 32 kb) Additional file 12:
**Information on the connectivity between projected maps.** (a) HMap: consensus map from Hamon et al. [43]; (b) TMap: consensus map from Tayeh et al. [49]; (c) number of common markers between HMap and TMap for each linkage group (LG); (d): Number of inversions of marker positions on each LG; (e) number of inversions of marker positions in confidence intervals of the quantitative trait loci (QTL) described by Hamon et al. [43]. (XLSX 11 kb) Additional file 13:
**Description of linkage disequilibrium (LD) block haplotypes in the pea-Aphanomyces collection.** (a) LD blocks are named as described in Additional file 11; (b) Each LD block is subdivided into marker haplotypes according to its allelic composition. Marker haplotypes are named with the LD block name followed by an Arabic numeral. Only haplotypes without missing values or heterozygous markers are shown; For each LD block: (c) list of markers significantly detected by genome-wide association in the LD block; (d) the first line is a list of the markers included in the LD block (detected markers in bold font and their markers in (LD) in plain font); (e) the second line is the genetic positions of the listed markers on the genetic map described in Additional file 11; (f) following lines are the pairwise LD *r* ^*2*^ values between each marker defined in the LD block and the marker detected by GWA; (g) For SNP markers, ‘AA’ and ‘BB’ are allele codes of Genome Studio analysis while for SSR markers, amplicon lengths are indicated. For each Aphanomyces resistance associated marker, enhancing allele(s) is in bold dark green font and unfavourable allele(s) in bold dark red font; (h) the first line is the list of the variables significantly associated with a marker in the LD block, variables are described as in Additional file 4; (i) next lines are *p-values* of the marker-trait associations; (j) grey or coloured lines are mean phenotypic values ± standard error for marker haplotypes carried by more than 5 % of the lines from the pea-Aphanomyces collection, letters indicate significantly different means (Tukey-HSD, α *<* 0.05); dark green and red haplotypes: favourable and unfavourable haplotypes of the LD block, respectively, as defined in the Materiel and Methods section of the manuscript. (XLSX 73 kb) Additional file 14:
**Marker haplotype composition of the pea-Aphanomyces collection.** (a) Name of marker haplotype carried by each line as described in Additional file 13, at each of the 14 consistent linkage disequilibrium (LD) blocks detected; dark green and red: favourable and unfavourable marker haplotypes, respectively, as defined in the Material and Methods section of the manuscript; (b) coordinates of each line of the collection on the first Multiple Factor Analysis principal component; the pea lines of the collection are ranked according to these coordinates; (c) Composition of each line in favourable and unfavourable haplotypes, is described in Additional file 13; (d) marker haplotypes with either a missing value or heterozygous score for at least one marker of a LD block. (XLSX 66 kb) Additional file 15:
**Annotation of markers in detected linkage disequilibrium (LD) blocks for putative candidate genes.** (a) LD block were constructed with markers detected by genome-wide association mapping and markers in LD with them, LD block are named according to Additional file 11; (b) genetic positions are estimated as described in Additional file 11; For each LD block:(c) traits associated with markers in the LD block; (d) Pairwise LD between each marker and the marker(s) detected by GWA in the LD block (detected marker in bold); (e) putative protein annotation underlying each marker based on comparative Blast X data from Tayeh et al. [49], see Methods section; (f) putative function of the protein in plant disease resistance based on the literature; (g) putative function of the protein in plant development based on the literature; (h) reference for protein function involved in plant disease resistance or plant development; (i) Pea cloned genes referred in literature; (j) extract of annotation data from Tayeh et al. [49], Blast X of marker sequence onto *P. sativum*, *M. truncatula*, *G. max* and *A. thaliana* proteins. (XLSX 131 kb) Additional file 16:
**Least square means (LSMeans) of phenotypic data in the pea-Aphanomyces collection after ANOVA.** Variables are coded as in Additional file 4. NA: missing data. (XLSX 71 kb) Additional file 17:
**Genotyping data of the pea-Aphanomyces collection used for the GWA analysis before filters and imputation.** NA: missing data. (a) Morphological gene genotyping data are coded “AA” when phenotypes are white flowers, normal leaves or smooth seeds for *A*, *Af* and *R* genes, respectively, and “BB” when they are colored flowers, afila leaves or wrinkled seeds; (b) PCR specific genotyping data are coded “AA” or “BB” when specific to the first or second allele; (c) SSR genotyping data are presented with the allele size (in bp); (d) SNP genotyping data are coded “AA” or “BB” when homozygous for the first or second allele, “AB” when heterozygous. (XLSX 7252 kb)

## Abbreviations

(c)DNA: (coding) deoxyribonucleic acid
ADI: aerial decline index
ADI1: first aerial decline index
ADI2: second aerial decline index
CC: controlled conditions
CI: confidence interval
DI: Dijon-Epoisses, France
DSP: Dark Skin Perfection (germplasm)
E: environment
EMMA: efficient mixed-model association
FLO1: number of calendar days to 50 % bloom
FLO2: number of calendar days to 100 % bloom
G: genotype
GAPIT: Genome Association and Prediction Integrated Tool
GSP: Groupement des sélectionneurs de pois protéagineux
GWA: genome-wide association
GxE: Genotype x environment interaction
HMap: consensus map from [43]
HT: plant height at 100 % bloom
INRA: Institut national de la recherche agronomique
KEN: Kendrick, USA
LD: linkage disequilibrium
LG: linkage group
LM: linear model
LRR: leucine rich repeat
LSMeans: least square means
MAF: minor allele frequency
MAGIC: multi-parent advanced generation inter-cross
MAS: marker assisted selection
mBonf: multiple-Bonferroni threshold
MFA: multiple factors analysis
MFA.Dim.1: first principal component of MFA
MFA.Dim.2: second principal component of MFA
MLMM: multi-locus mixed model
NAM: nested association mapping
NBS: nucleotide binding site
NGS: next generation sequencing
NIL: near-isogenic line
PC: principal component
PCA: principal components analysis
PCR: polymerase chain reaction
QTL: quantitative trait locus
*R*: replicate
REN: Rennes-Le Rheu, France
RI: Riec-sur-Belon, France
RIL: recombinant inbred line
RIPE: number of calendar days to 100 % dried plants
RRI: root rot index
SGR: stay green gene
SNP: single nucleotide polymorphism
SSR: simple sequence repeats
THMap: consensus map from projection of HMap onto TMap
TMap: consensus map from [49]
UPGMA: unweighted pair group method with arithmetic mean
USA: United States of America
USDA: United States Department of Agriculture
