# Supplementary figures and images for: Genome-wide association mapping of partial resistance to Aphanomyces euteiches in pea
Source: BMC Genomics. 2016 Feb 20;17:124. doi: 10.1186/s12864-016-2429-4 (PMC4761183; doi:10.1186/s12864-016-2429-4)

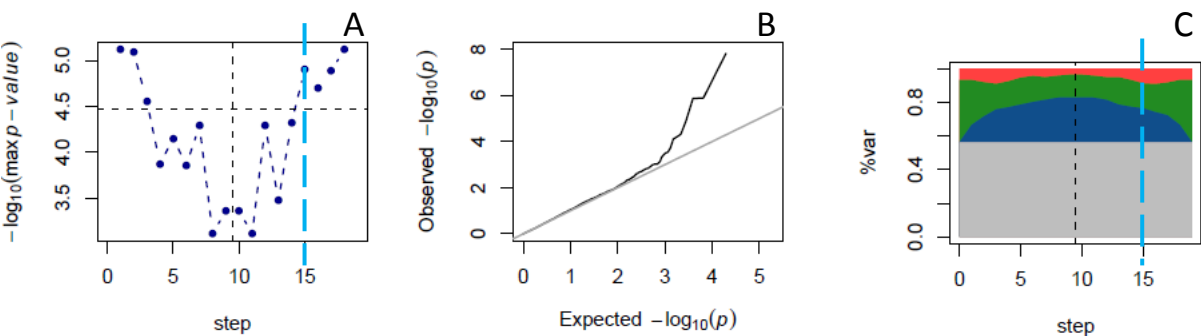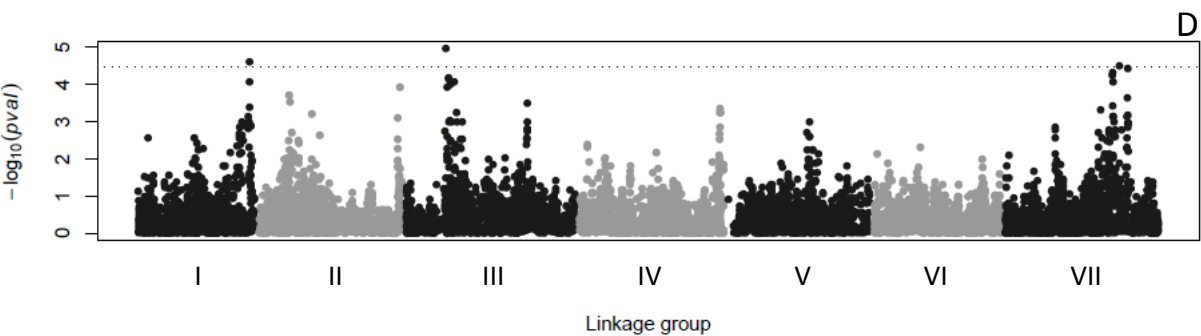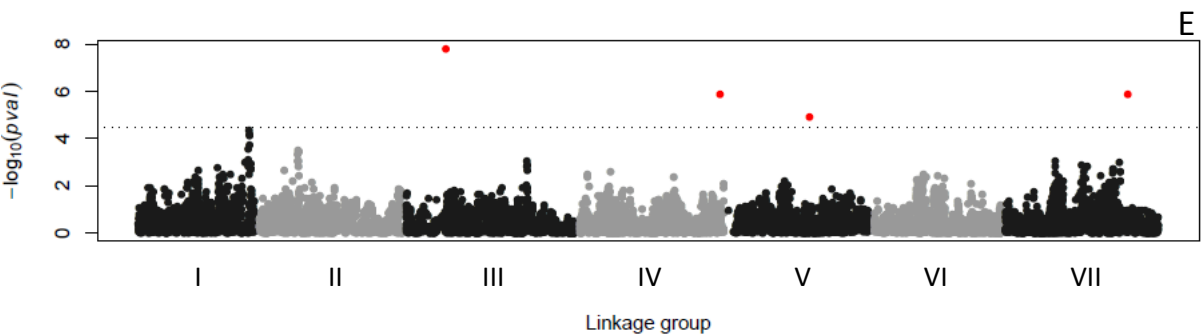

Supplement: Additional file 3: — Example of MLMM R package plot outputs for analysis of the MFA.Dim.1 variable. (A): Bonferroni correction of the highest marker p-value for each step of the forward and backward analysis. Optimal step (dashed blue vertical line) is determined as the largest stepwise mixed model regression in which all cofactors have –log10(p-value) above the mBonf threshold (dashed black horizontal line). (B): QQ-plot at the optimal step. (C): Partition of variance for each forward and backward step. Variance explained by population structure (PCA; grey); all markers used as cofactors (blue); Kinship matrix (green) and unexplained variance (= missing heritability; red). The dashed blue vertical line represents the optimal step according to mBonf threshold. (D) Genome-wide Manhattan plot without marker cofactors. The dotted line represents the multiple-Bonferroni threshold above which markers are considered as significant. (E): Genome-wide Manhattan plot at the optimal step with marker cofactors (4 red dots). The dotted line represents the multiple-Bonferroni threshold above which markers are considered as significant. (PDF 148 kb) [file 12864_2016_2429_MOESM3_ESM.pdf]

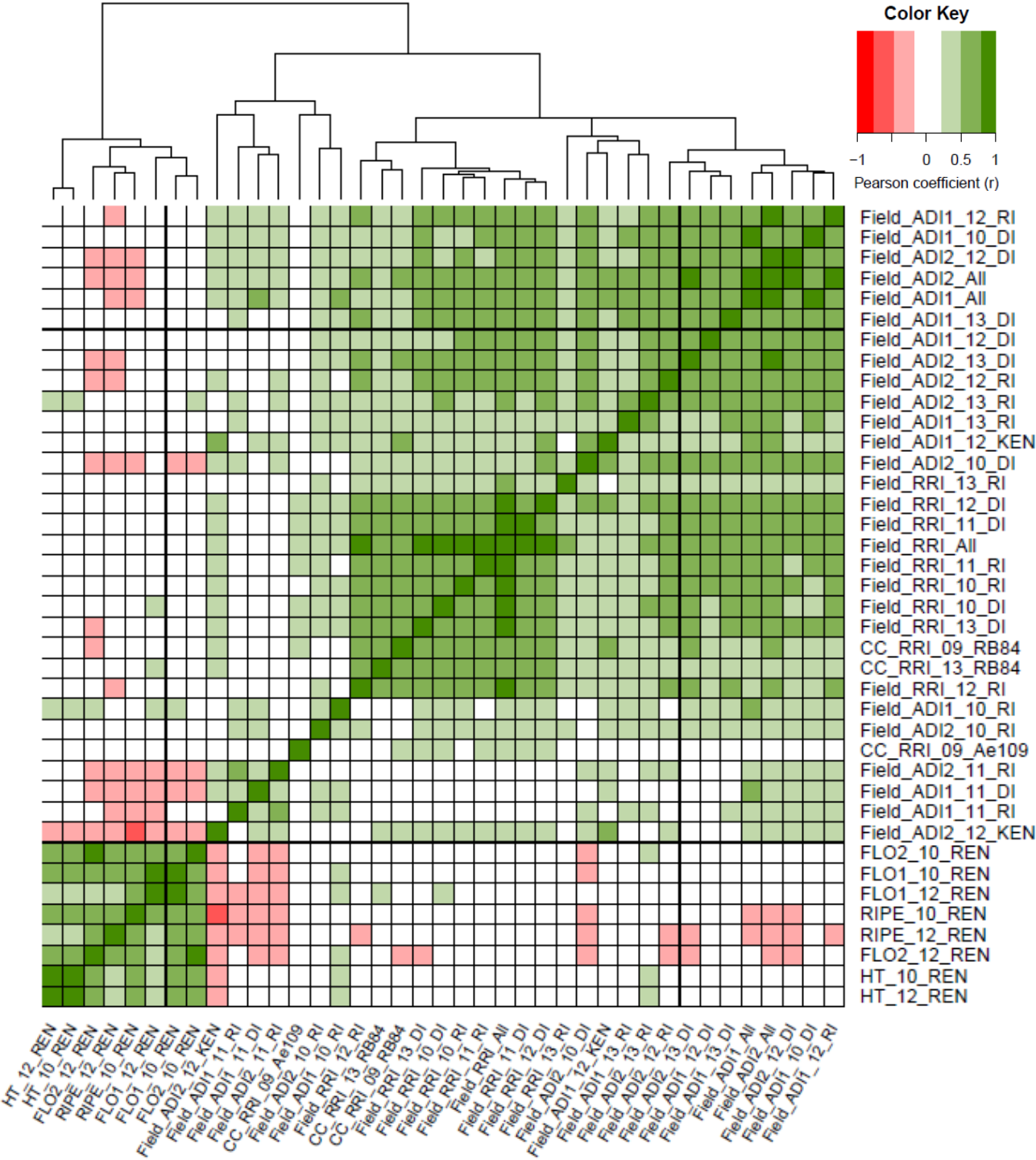

Supplement: Additional file 6: — Clustered heatmap of correlation coefficients between disease resistance, earliness and height variables data. Variables are coded as described in Additional file 4. Only correlations with a p-value < 0.05 are shown. Colours represent level of correlation, coded as follow: dark red: r < −0.8; medium red: −0.8 < r < −0.5; light red: −0.5 < r < −0.2; white: −0.2 < r < 0.2 or p-value > 0.05; light green: 0.2 < r < 0.5; medium green: 0.5 < r < 0.8; dark green: r > 0.8. Clustering is based on the UPGMA method. (PDF 48 kb) [file 12864_2016_2429_MOESM6_ESM.pdf]

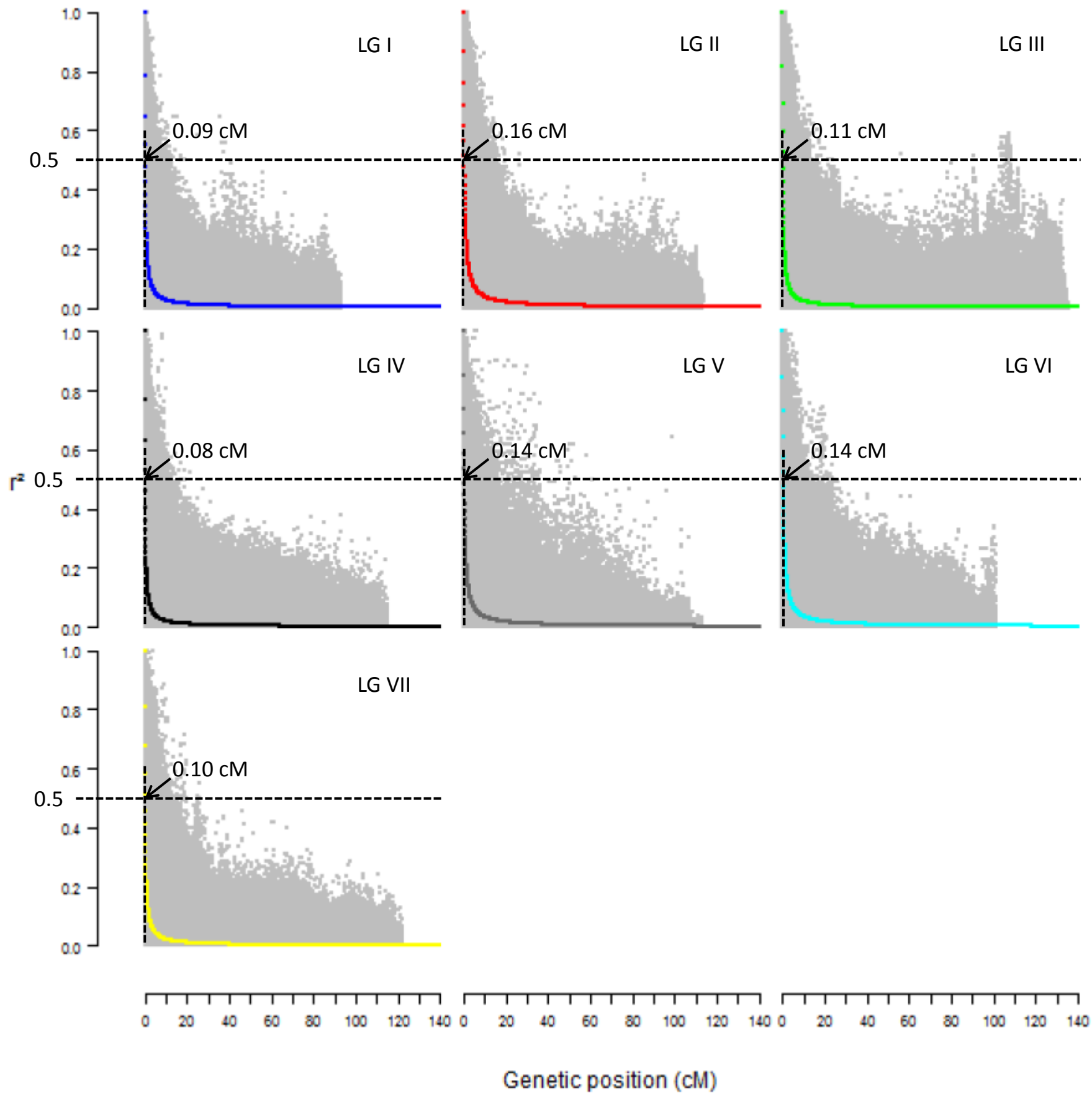

Supplement: Additional file 8: — Linkage disequilibrium (LD) decay in the pea-Aphanomyces collection. Coloured curves represent the estimated LD decay for each linkage group (LG). Dashed horizontal lines represent half of the maximum LD value (LD threshold=0.5). Arrows represent the LD decay rate, as the estimated genetic distance (cM) to drop to the LD threshold on each LG. (PDF 157 kb) [file 12864_2016_2429_MOESM8_ESM.pdf]

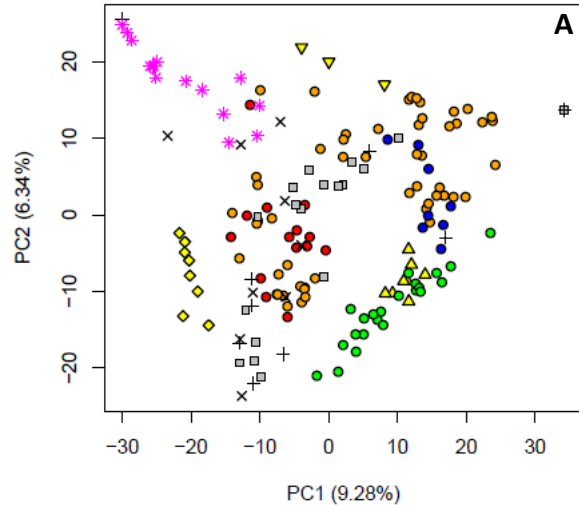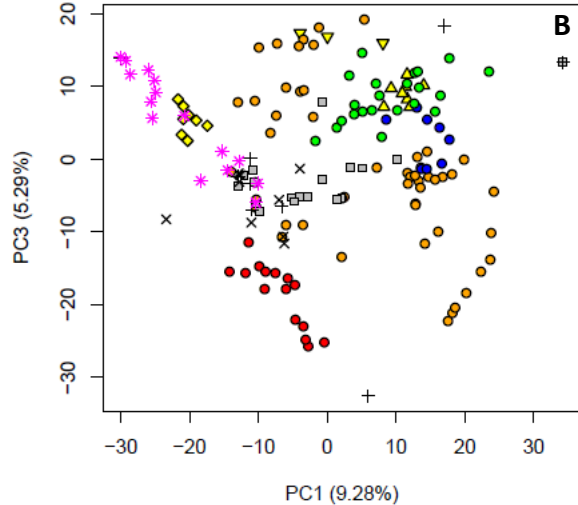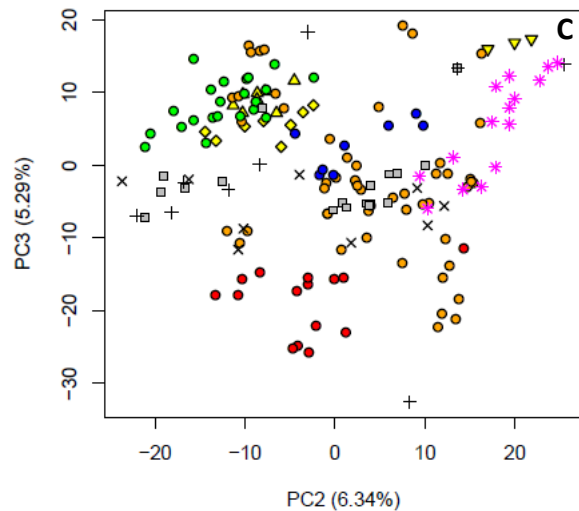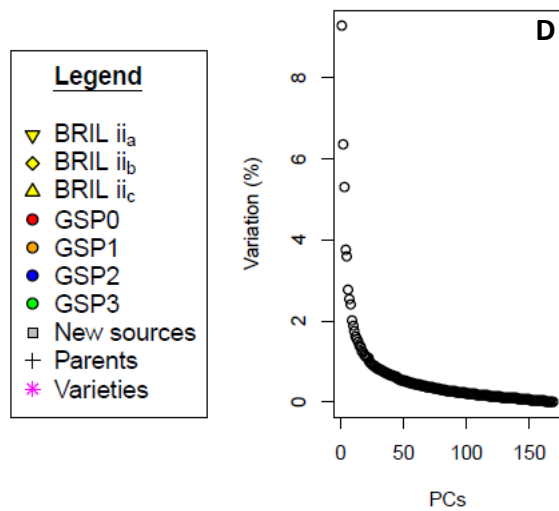

Supplement: Additional file 9: — Population structure of the pea-Aphanomyces collection based on Principal Component Analysis (PCA). PCA from GAPIT R package [75] based on 2937 markers. Distributions of pea lines of the collection are represented on the first three principal components (A–C), which explain a total of 20.91 % of inertia (D). Categories of pea lines are described in Fig. 1 and Additional file 1. (D) Inertia contribution of each principal component (from PC1 to PC169). (PDF 151 kb) [file 12864_2016_2429_MOESM9_ESM.pdf]

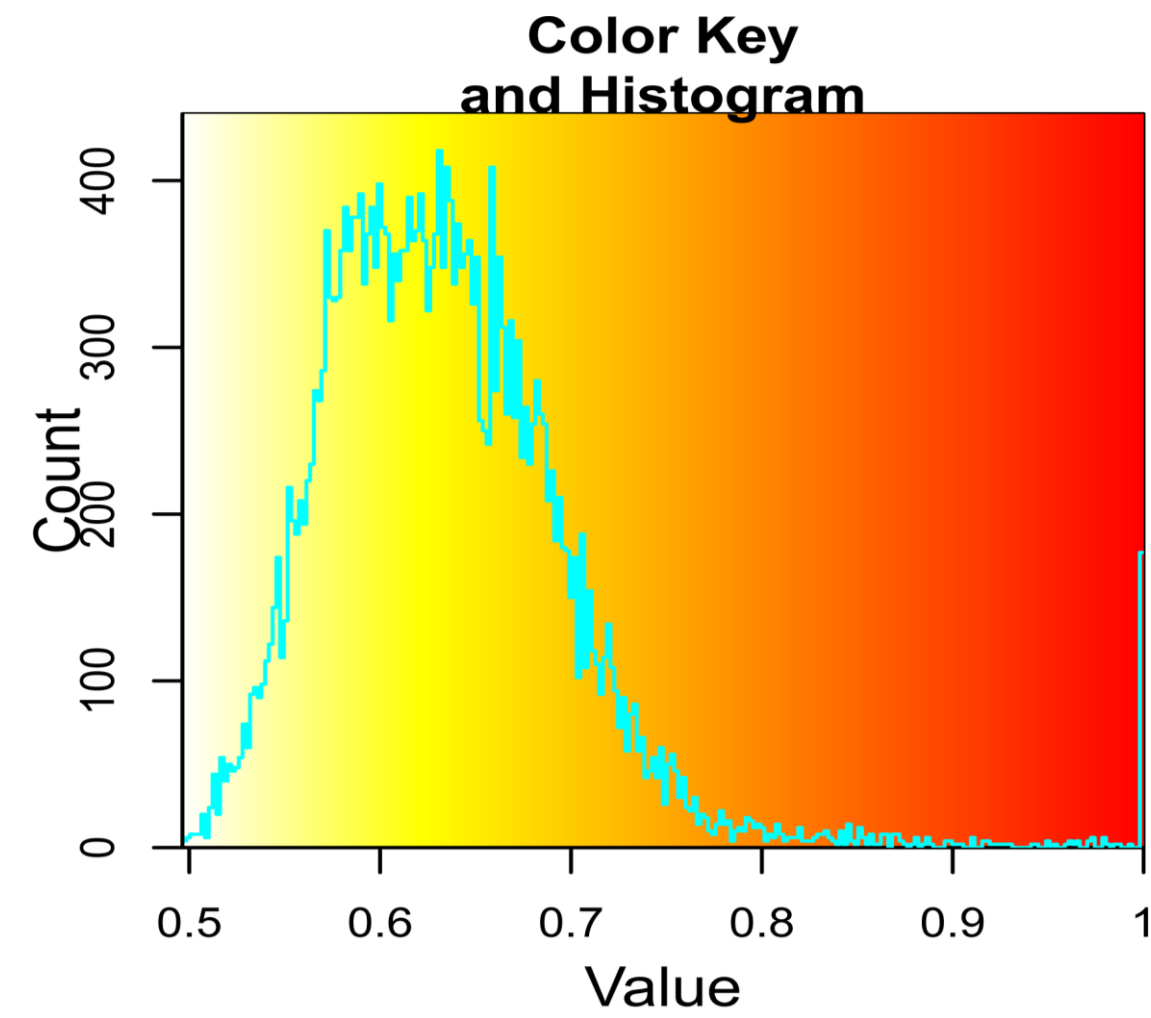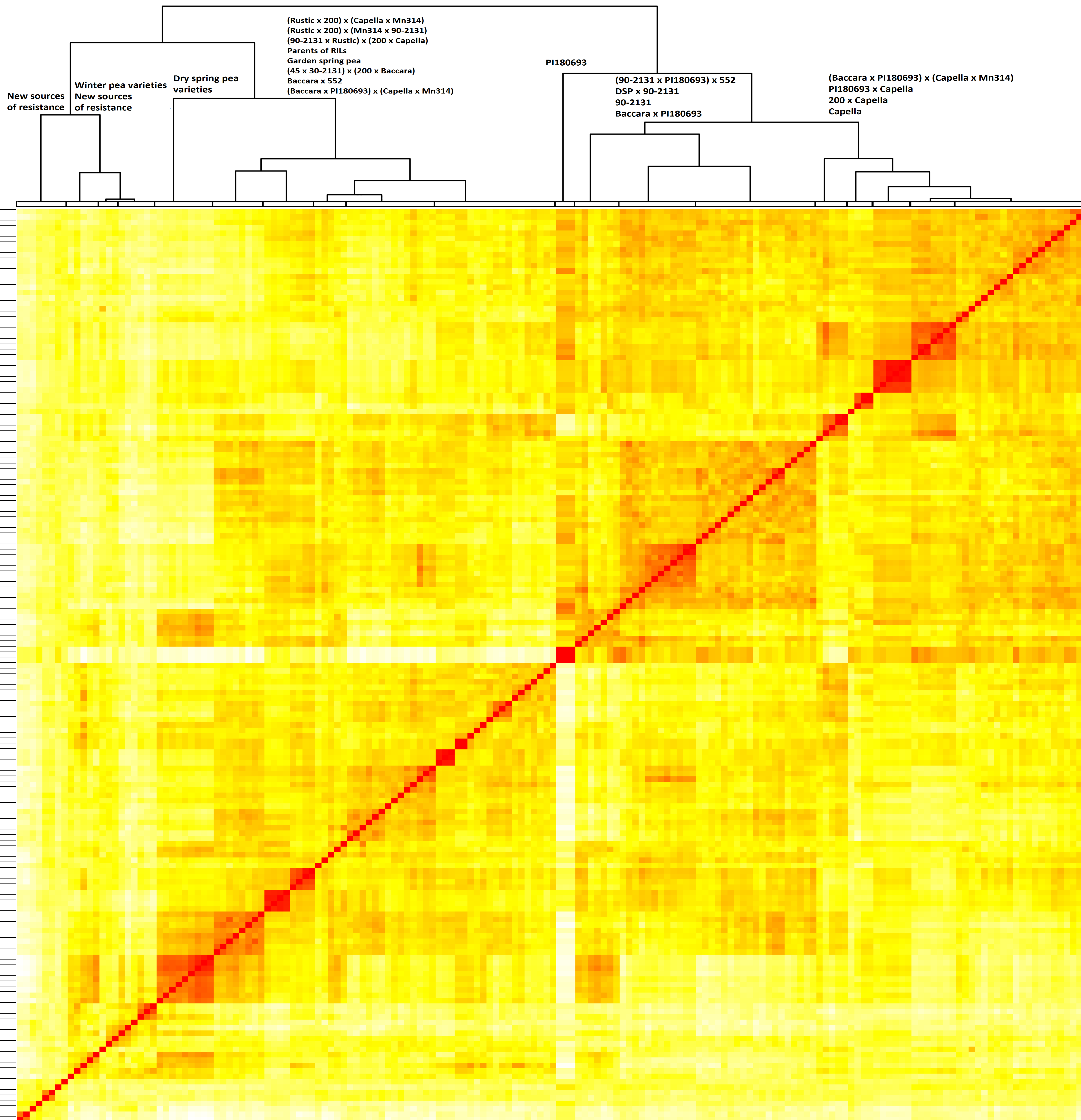

Supplement: Additional file 10: — Clustered heatmap of the Kinship matrix. Kinship matrix from the GAPIT R package [75] based on 2937 SNP markers. Clustering is based on the UPGMA method. Colours represent the degree of relationship between two given lines. Pea line information is described as in Additional file 1. Lines sharing same pedigree or end use or sowing type are well clustered thus the Kinship matrix efficiently represents the relationships between individuals. (PDF 1253 kb) [file 12864_2016_2429_MOESM10_ESM.pdf]
