# Supplementary material for: Genome-wide association mapping of partial resistance to Aphanomyces euteiches in pea
Source: BMC Genomics. 2016 Feb 20;17:124. doi: 10.1186/s12864-016-2429-4 (PMC4761183; doi:10.1186/s12864-016-2429-4)
Supplement: Additional file 5: — Frequency distribution of least square means obtained for Aphanomyces resistance, earliness and plant height in the pea-Aphanomyces collection. Least square means were obtained from analysis of variance for three Aphanomyces resistance traits (Root Rot Index, first and second Aerial Decline Indexes, coded RRI, ADI1 and ADI2, respectively), three earliness traits (dates to 50 % bloom, 100 % bloom and 100 % dried plants, coded FLO1, FLO2 and RIPE, respectively) and plant height (HT). Aphanomyces resistance traits were assessed over nine infested field environments and against two reference strains of A. euteiches (RB84 and Ae109) in controlled conditions. Earliness and height were assessed in two healthy environments. n: total number of pea lines assessed; m: mean ± standard deviation of the pea-Aphanomyces collection; h2: mean-based heritability. (PDF 38 kb) [file 12864_2016_2429_MOESM5_ESM.pdf]

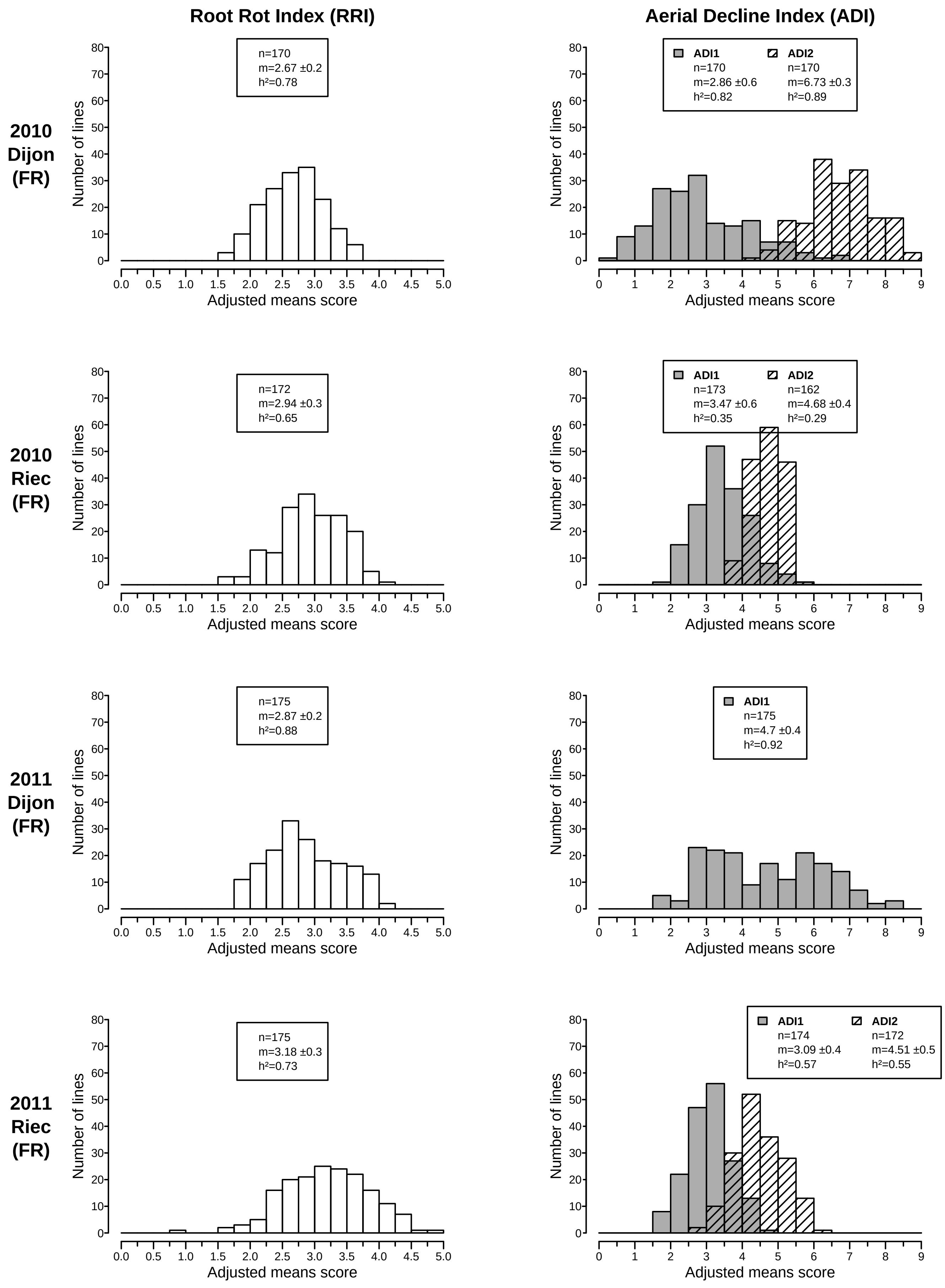
Root Rot Index (RRI)
Aerial Decline Index (ADI)
2010 Dijon (FR)
n=170
m=2.67 ±0.2
h²=0.78
ADI1
n=170
m=2.86 ±0.6
h²=0.82
ADI2
n=170
m=6.73 ±0.3
h²=0.89
2010 Riec (FR)
n=172
m=2.94 ±0.3
h²=0.65
ADI1
n=173
m=3.47 ±0.6
h²=0.35
ADI2
n=162
m=4.68 ±0.4
h²=0.29
2011 Dijon (FR)
n=175
m=2.87 ±0.2
h²=0.88
ADI1
n=175
m=4.7 ±0.4
h²=0.92
2011 Riec (FR)
n=175
m=3.18 ±0.3
h²=0.73
ADI1
n=174
m=3.09 ±0.4
h²=0.57
ADI2
n=172
m=4.51 ±0.5
h²=0.55
Number of lines
Adjusted means score
0
10
20
30
40
50
60
70
80
0.0
0.5
1.0
1.5
2.0
2.5
3.0
3.5
4.0
4.5
5.0
1
2
3
4
5
6
7
8
9

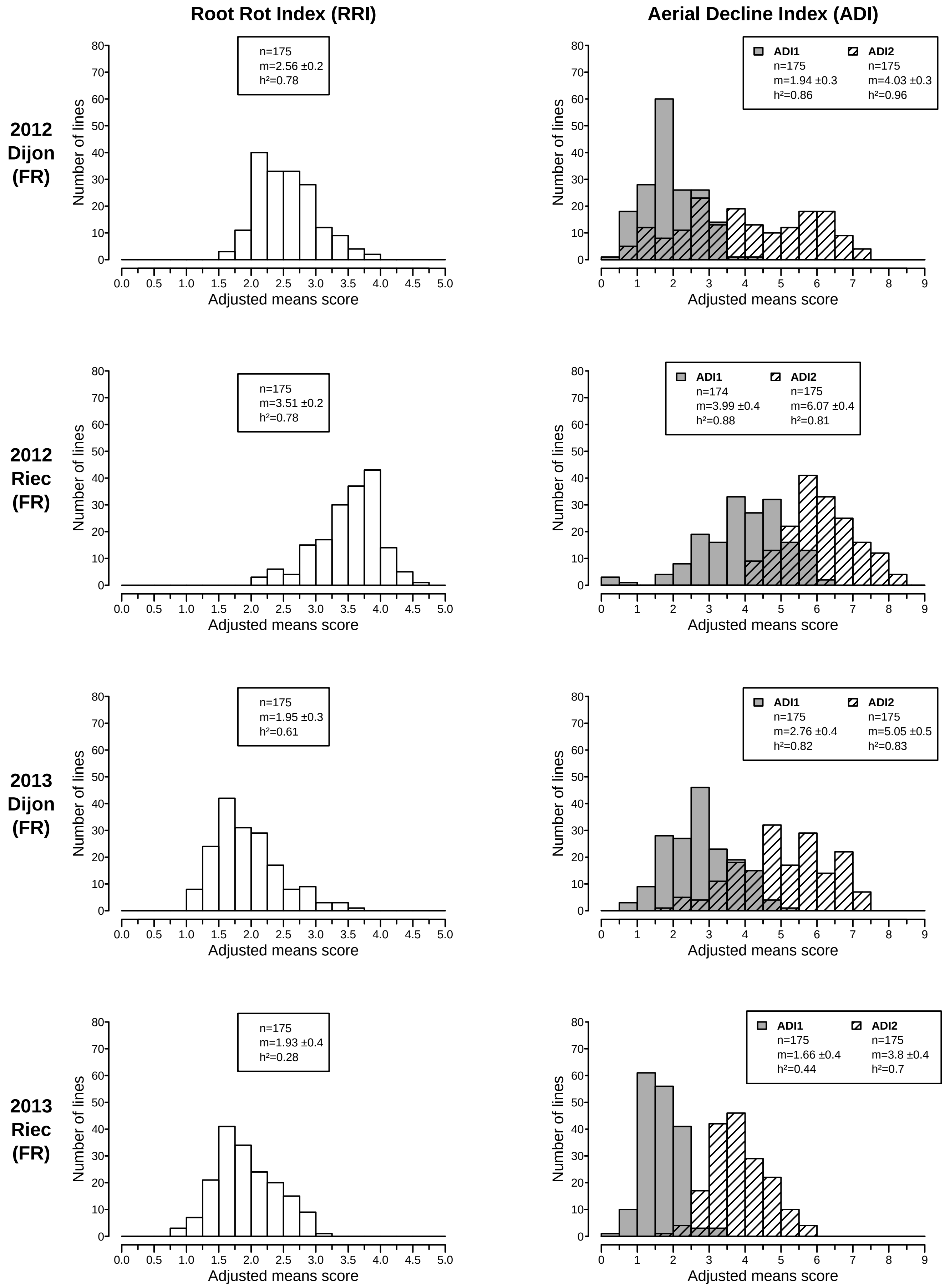
Root Rot Index (RRI)
Aerial Decline Index (ADI)
2012 Dijon (FR)
n=175
m=2.56 ±0.2
h²=0.78
ADI1
n=175
m=1.94 ±0.3
h²=0.86
ADI2
n=175
m=4.03 ±0.3
h²=0.96
2012 Riec (FR)
n=175
m=3.51 ±0.2
h²=0.78
ADI1
n=174
m=3.99 ±0.4
h²=0.88
ADI2
n=175
m=6.07 ±0.4
h²=0.81
2013 Dijon (FR)
n=175
m=1.95 ±0.3
h²=0.61
ADI1
n=175
m=2.76 ±0.4
h²=0.82
ADI2
n=175
m=5.05 ±0.5
h²=0.83
2013 Riec (FR)
n=175
m=1.93 ±0.4
h²=0.28
ADI1
n=175
m=1.66 ±0.4
h²=0.44
ADI2
n=175
m=3.8 ±0.4
h²=0.7
Number of lines
Adjusted means score

Root Rot Index (RRI) | **Aerial Decline Index (ADI)**

2012 Kendrick (USA)

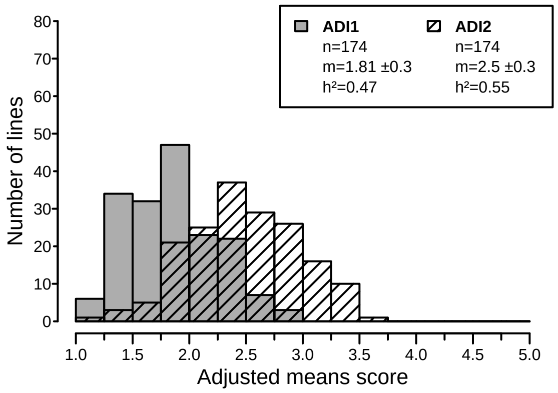

2009 Ae109 (CC)

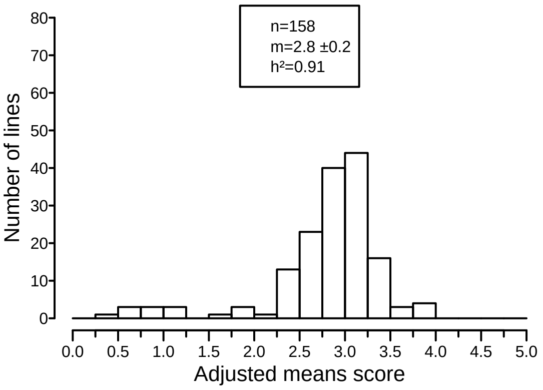

2009 RB84 (CC)

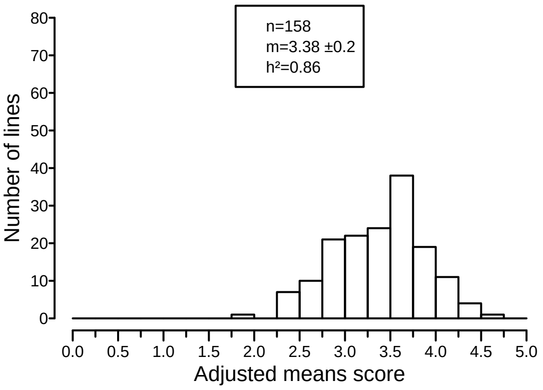

2013 RB84 (CC)

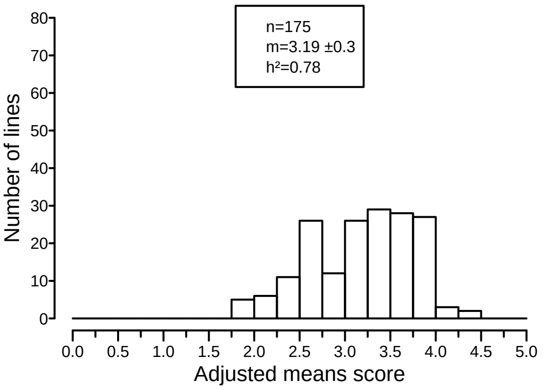

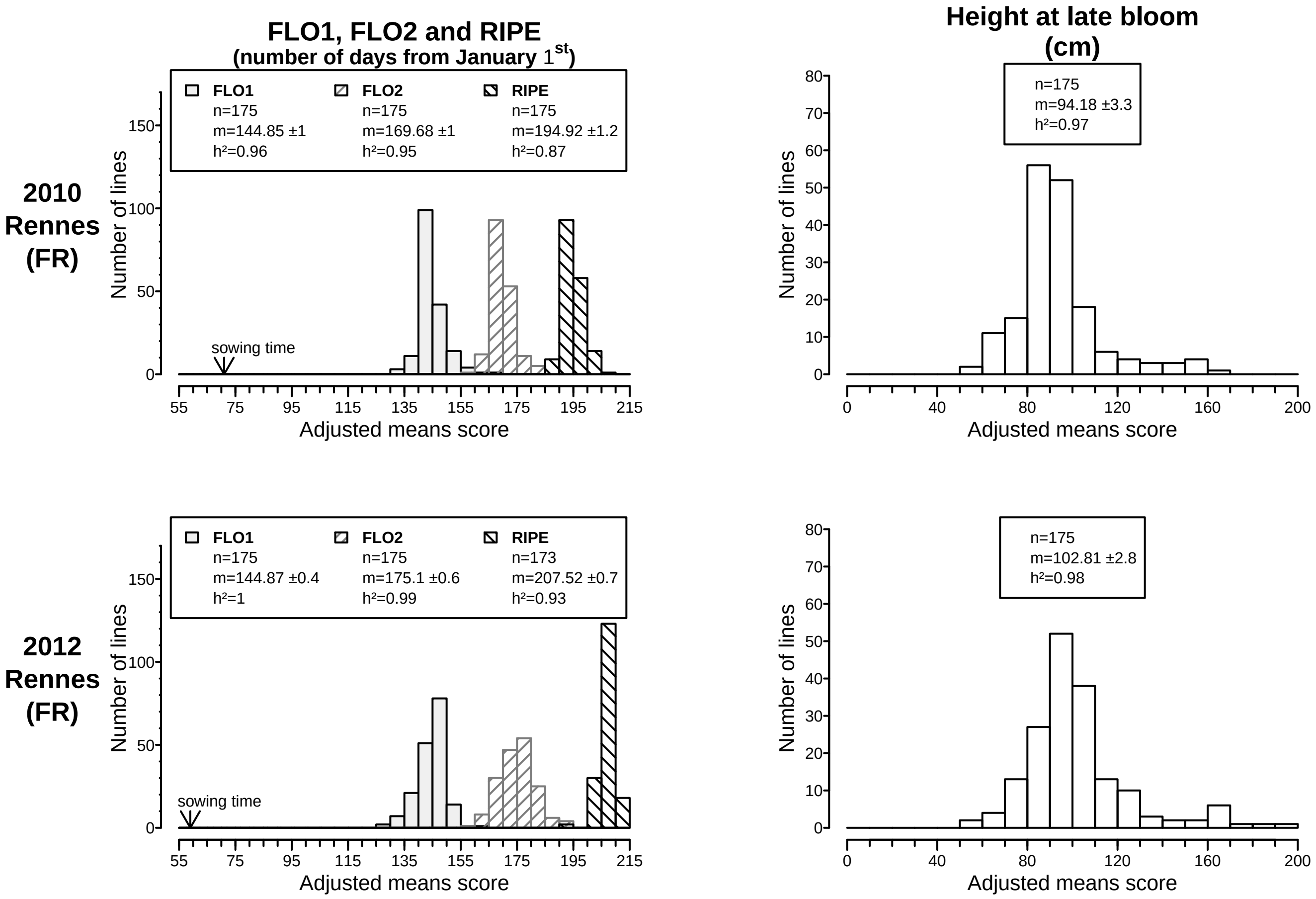
FLO1, FLO2 and RIPE
(number of days from January 1st)
Height at late bloom
(cm)
2010
Rennes
(FR)
2012
Rennes
(FR)
FLO1
n=175
m=144.85 ±1
h²=0.96
FLO2
n=175
m=169.68 ±1
h²=0.95
RIPE
n=175
m=194.92 ±1.2
h²=0.87
sowing time
Number of lines
Adjusted means score
n=175
m=94.18 ±3.3
h²=0.97
FLO1
n=175
m=144.87 ±0.4
h²=1
FLO2
n=175
m=175.1 ±0.6
h²=0.99
RIPE
n=173
m=207.52 ±0.7
h²=0.93
n=175
m=102.81 ±2.8
h²=0.98
